# Enhanced kinase translocation reporters for simultaneous real-time measurement of PKA, ERK, and calcium

**DOI:** 10.1016/j.jbc.2025.108183

**Published:** 2025-01-13

**Authors:** Shang-Jui Tsai, Yijing Gong, Austin Dabbs, Fiddia Zahra, Junhao Xu, Aleksander Geske, Michael J. Caterina, Stephen J. Gould

**Affiliations:** 1 Department of Neurosurgery, Johns Hopkins University School of Medicine, Baltimore, Maryland, USA; 2 Department of Neuroscience, Johns Hopkins University School of Medicine, Baltimore, Maryland, USA; 3 Department of Biological Chemistry, Johns Hopkins University School of Medicine, Baltimore, Maryland, USA

**Keywords:** protein kinase, biosensor, live cell imaging, nuclear pore, nuclear import, nuclear export, signal transduction

## Abstract

Kinase translocation reporters (KTRs) are powerful tools for single-cell measurement of time-integrated kinase activity but suffer from restricted dynamic range and limited sensitivity, particularly in neurons. To address these limitations, we developed enhanced KTRs (eKTRs) for PKA and extracellular signal–regulated kinase (ERK) by (i) increasing KTR size, which reduces the confounding effect of KTR diffusion through the nuclear pore and (ii) modulating the strength of the bipartite nuclear localization signal in their kinase sensor domains, to ensure that the relative distribution of the KTR between the nucleus and cytoplasmic is determined by active nuclear import, active nuclear export, and relative activity of their cognate kinase. The resultant sets of ePKA-KTRs and eERK-KTRs display high sensitivity, broad dynamic range, and cell type–specific tuning. Moreover, co-expression of optically separable ePKA-KTRs and eERK-KTRs allowed us to simultaneously monitor the activation and inhibition of PKA and ERK, together with calcium levels, in live cells. These eKTRs responded as expected to direct agonists and inhibitors, and also confirmed that crosstalk between the PKA and ERK pathways is highly unbalanced, with the activation of PKA suppressing ERK activity, while activation of ERK induces PKA activity. Taken together, our findings highlight the importance of KTR size and bipartite nuclear localization signal strength to KTR sensitivity and dynamic range, show that different cell types require different eKTRs, and identify ePKA-KTR1.4 and eERK-KTR1.2 as particularly well-suited for monitoring PKA and ERK in primary sensory neurons.

Protein kinase signaling pathways play critical roles in biology, and PKA and ERK have long been recognized as particularly important (1, 2, 3, 4). PKA is a key effector of the second messenger cAMP, facilitating the transmission of key metabolic and physiological signals, such as the flight-or-flight hormone adrenaline, the fasting hormone glucagon, and multiple inflammatory signals (5, 6, 7). In contrast, ERK is a key effector of growth factor signaling and lipid signaling, exemplified by epidermal growth factor (EGF)-mediated activation of EGF receptors and the Ras/Raf/MEK/ERK pathway, as well as by diacylglycerol-mediated activation of the PKC/MEK/ERK pathway (4, 8, 9). A variety of fluorescent protein (FP)–based kinase biosensors have been developed for monitoring PKA and ERK activities in living cells, and these have yielded important new insights into the PKA and ERK signaling pathways (10, 11, 12, 13, 14). These fluorescent protein biosensors range in their timescale of detection, from nonintegrative sensors that detect changes in kinase activity on the seconds-to-minutes timescale, to transcription-based sensors that report kinase activities on the timescale of hours-to-days (15, 16, 17, 18, 19, 20, 21, 22, 23, 24, 25, 26). Between these two temporal extremes, the kinase translocation reporters (KTRs) provide a mesoscale, temporally-integrative readout of kinase activation and inhibition at the minutes-to-hour timescale that corresponds to many physiological processes (27, 28).

As originally conceived (27), KTRs are comprised of an FP fused to a kinase sensor domain that has four functional components: (i) a kinase-binding site, (ii) a bipartite nuclear localization signal (bNLS), (iii) a nuclear export signal (NES), and (iv) a pair of kinase phosphorylation sites, one in the central region of the bNLS and the other positioned between the bNLS and NES (27). Theoretically, this architecture allows the KTR to continuously engage the cell’s nuclear import machinery and nuclear export machinery, with the KTR’s relative distribution between nucleus and cytoplasm determined by the relative strengths of their bNLS and NES, which are modulated by kinase-mediated phosphorylation of their sensor domain. Ideally, the activity of the bNLS and NES are in a reciprocally regulated tension, with the bNLS dominating over the NES when kinase activity is low, resulting in the nuclear accumulation of the KTR, while kinase-mediated phosphorylation of the sensor domain weakens the bNLS so much that the NES dominates over the bNLS, leading to the KTR accumulation in the cytoplasm. As a result, KTRs allow researchers to monitor kinase activity using simple, widefield fluorescence microscopy, with kinase activity proportional to the KTR’s relative fluorescence in the cytoplasm and nucleus, represented numerically by the KTR’s cytoplasm/nucleus (C/N) ratio. Furthermore, KTRs are amenable to modeling studies that can glean even more information from their kinase-dependent changes in distribution (29).

Given that KTRs are integrative biosensors that operate at biologically meaningful timescales, their optimization is of high priority. However, efforts at improving KTR performance characteristics have so far been limited to identification of kinase-binding site mutation (R7A) in the ERK KTR that improves its specificity for ERK (30) and a high-throughput, barcoding strategy for hypothesis-free identification of KTR sensor domain sequences (31). Using PKA-KTR as a proof-of-principle example, we show here that KTRs can be improved significantly by (i) increasing KTR size above ∼40 kDa, to ensure that their C/N ratios are not confounded by free diffusion through the nuclear pore and (ii) varying the sequence and strength of their bNLS strength, to ensure that the bNLS dominates over the NES in unstimulated cells, but is weaker than its NES in kinase-activated cells. Furthermore, we demonstrate these advances for both PKA and ERK, describe sets of enhanced KTRs (eKTRs), and demonstrate that ePKA-KTR1.4 and eERK-KTR1.2 allow researchers to monitor PKA and ERK activities in primary sensory neurons.

## Results

### Tuning bNLS strength to improve the sensitivity and dynamic range of PKA-KTR1

**Figure 1:**
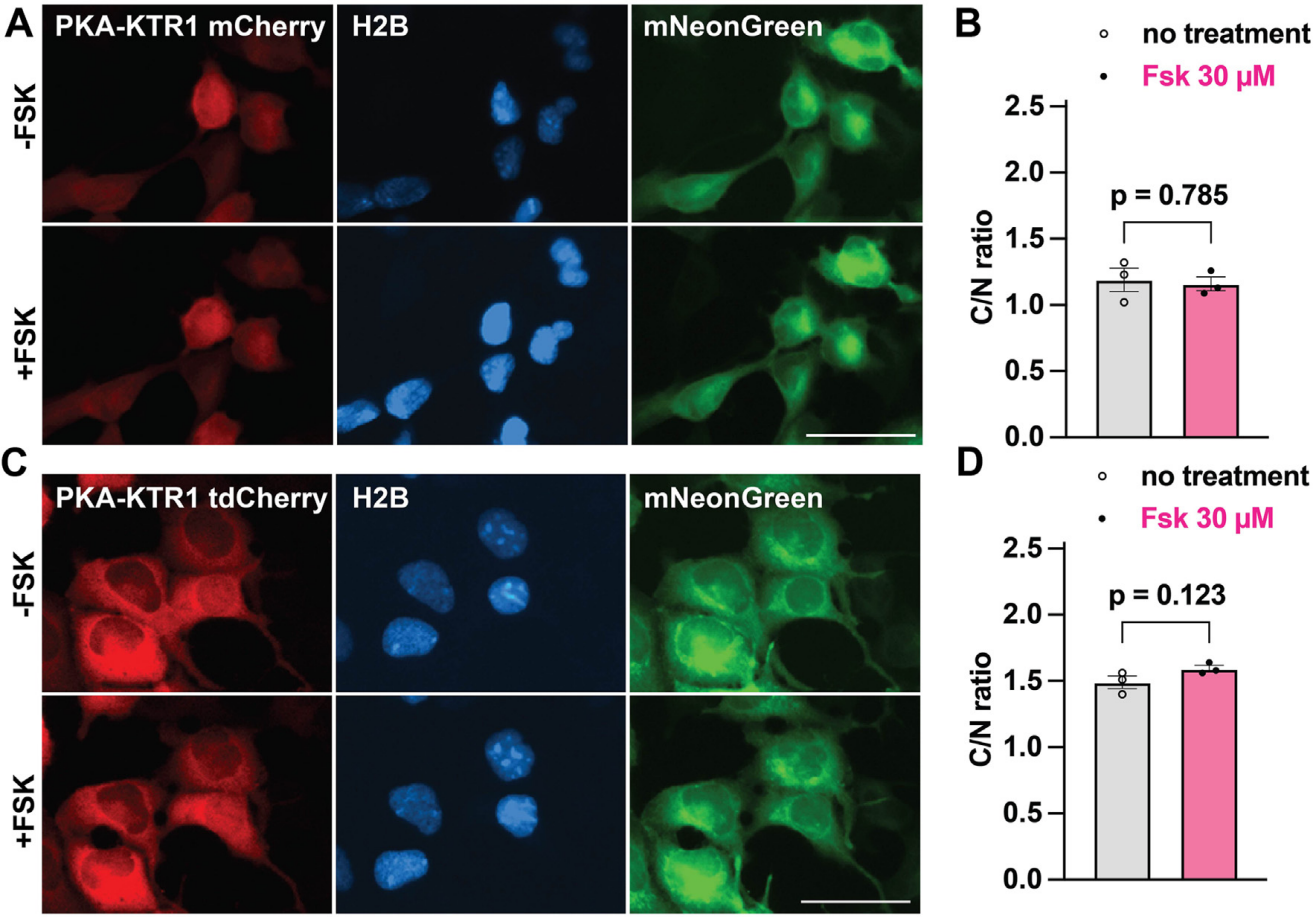
**The bNLS in PKA-KTR is too weak to direct proteins to the nucleus.***A*, representative fluorescence micrographs of HEK293 cells expressing the (*red*) PKA-KTR1/mCherry, (*blue*) H2B/mTagBFP2, and (*green*) mNeonGreen, grown in the absence of Fsk (−FSK) or after 30 min exposure to 30 μM forskolin (+FSK). Bar represents 50 μM. *B*, bar graph showing C/N ratios of PKA-KTR1/mCherry (*gray*) prior to the addition of Fsk or (*pink*) 30 min after the addition of Fsk. *C*, representative fluorescence micrographs of HEK293 cells expressing the (*red*) PKA-KTR1/tdCherry, (*blue*) H2B/mTagBFP2, and (*green*) mNeonGreen, grown in the absence of Fsk (−FSK) or after 30 min exposure to 30 μM forskolin (+FSK). Bar represents 50 μM. *D*, bar graph showing C/N ratios of PKA-KTR1/tdCherry (*gray*) prior to the addition of Fsk or (*pink*) 30 min after the addition of Fsk. All data in this figure are from a minimum of three independent biological replicates, the data for all bar graphs came from the analysis of independent groups of cells, the error bars reflect the SEM, and *p* values were calculated by two-tailed *t* test.

The original description of KTR technology described a KTR for PKA, PKA-KTR (27), which we refer to as PKA-KTR1. To measure PKA activity in live cells, we built a plasmid that expressed the kinase sensor domain of PKA-KTR1 fused to the N-terminus of mCherry, transfected it into HEK293 cells, and incubated these cells for 30 min in medium containing vehicle or forskolin (Fsk; at 30 uM), which activates adenylate cyclase, increases cAMP concentration, and activates PKA. However, when we examined these cells by fluorescence microscopy, we found that PKA-KTR1/mCherry was distributed equally between the nucleus and cytoplasm and that Fsk had no effect on its relative distribution between the cytoplasm and nucleus (Fig. 1*A*). Furthermore, when we measured the relative distribution of PKA-KTR1/mCherry fluorescence intensity over the nucleus and cytoplasm (defined by a nuclear mTagBFP2 and cytoplasmic mNeonGreen), across a minimum of 10 cells, from a minimum of three regions-of-interest (ROIs) and a minimum of three independent trials, we found no change in the C/N ratio of PKA-KTR1/mCherry (Fig. 1*B*), even though HEK293 cells are known to support Fsk-induced activation of PKA (32, 33).

The observation that PKA-KTR1/mCherry failed to show any enrichment in the nucleus suggests that the bNLS in the PKA-KTR1 kinase sensor domain is too weak to drive nuclear import. To test this idea more directly, we appended it to the N-terminus of tdCherry, a tandem dimer of mCherry, creating a PKA-KTR1/tdCherry fusion protein of 64 kDa. The size-dependence of protein diffusion through the nuclear pore is such that it typically allows for rapid diffusion of proteins smaller than ∼40 kDa, whereas proteins larger than ∼60 kDa typically show a strong accumulation where they are made, in the cytoplasm (34, 35, 36, 37, 38). Thus, if the bNLS in the PKA-KTR1 sensor domain has strong NLS activity, we would expect PKA-KTR1/tdCherry to accumulate in the nucleus. However, when we expressed PKA-KTR1/tdCherry in HEK293 cells, we found that it accumulated in the cytoplasm and had a high C/N ratio of ∼1.5 (Fig. 1, *C* and *D*). Furthermore, exposing these cells to Fsk had no effect on the C/N ratio of PKA-KTR1/tdCherry.

**Figure 2:**
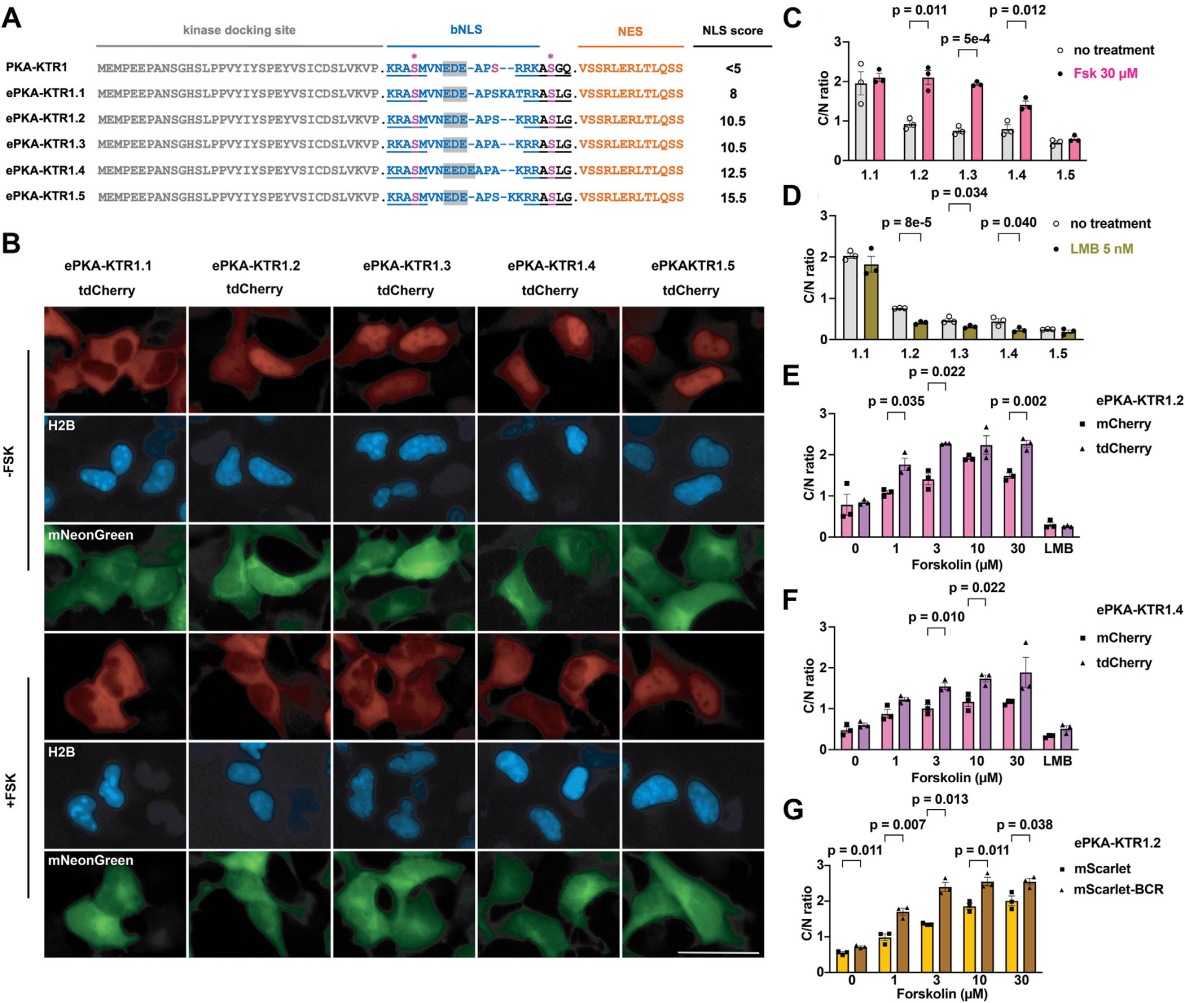
**Tuning NLS strength and KTR size improves PKA-KTR sensitivity and dynamic range.***A*, amino acid sequences of PKA-KTR sensor domains, showing the kinase docking site, bNLS, PKA phosphorylation sites (in *pink*, with asterisk), and NES, with predicted NLS strength scores listed to the *right*. Bar represents 50 μm. *B*, representative fluorescence microscopy images of HEK293 cells expressing the (*red*) ePKA-KTR1.1, 1.2, 1.3, 1.4, and 1.5 proteins, (*blue*) H2B/mTagBFP2, and (*green*) mNeonGreen, grown in the absence of Fsk (−FSK) or after 30 min exposure to 30 μM forskolin (+FSK). *C*, bar graph showing C/N ratios of ePKA-KTR1.1, 1.2, 1.3, 1.4, and 1.5/tdCherry proteins (*gray*) prior to the addition of Fsk or (*pink*) 30 min after the addition of Fsk. *D*, C/N ratios of ePKA-KTR1.1, 1.2, 1.3, 1.4, and 1.5/tdCherry (*gray*) prior to the addition of leptomycin B or (*green*) after 30 min incubation in leptomycin B. *E*, bar graphs comparing the C/N ratios of ePKA-KTR1.2/mCherry (*pink*) and ePKA-KTR1.2/tdCherry (*purple*) following 30 min incubation in 0, 1, 3, 10, or 30 μM Fsk, or 5 nM leptomycin B (LMB). *F*, bar graphs comparing the C/N ratios of (*pink*) ePKA-KTR1.4/mCherry and (*purple*) ePKA-KTR1.4/tdCherry following 30 min incubation in 0, 1, 3, 10, or 30 μM Fsk, or 5 nM leptomycin B (LMB). *G*, bar graphs comparing the C/N ratios of ePKA-KTR1.2/mScarlet (*gold*) or ePKA-KTR1.2/mScarlet/BCR (*brown*) following 30 min incubation in 0, 1, 3, 10, or 30 μM Fsk, or 5 nM leptomycin B (LMB). All data in this figure are from a minimum of three independent biological replicates, the data for all bar graphs came from the analysis of independent groups of cells, the error bars reflect the SEM, and *p* values were calculated by two-way ANOVA.

Our conclusion from these experiments is that the bNLS in the kinase sensor domain of PKA-KTR1 was too weak for it to function as a PKA sensor domain. In support of this hypothesis, the cNLS Mapper algorithm (39), which predicts the relative strength of NLS sequences, assigned the bNLS of the PKA-KTR1 sensor domain a very low score, <5 (Fig. 2*A*). To see if increasing the bNLS strength could convert PKA-KTR1 into a better sensor of PKA activity, we generated variants of the bNLS sequence of that have higher cNLS Mapper scores, ranging from 8 to 15.5 (Fig. 2*A*). Next, we generated plasmids designed to express tdCherry proteins carrying these enhanced PKA (ePKA) kinase sensor domains at their N-terminus, transfected these plasmids into HEK293 cells, and examined the subcellular distribution of each ePKA KTR1 ± 30 μM Fsk (30 min incubation). At baseline, the subcellular distribution of each ePKA-KTR bore a rough correlation to their predicted strength of their NLS, with ePKA-KTR1.1 (cNLS Mapper score of 8) having the highest C/N ratio, ePKA-KTR1.5 (cNLS Mapper score of 15.5) having the lowest C/N ratio, and ePKA-KTR1.2-1.4 having intermediate C/N ratios (cNLS Mapper scores of 10.5–12.5) (Fig. 2, *B* and *C*). Furthermore, we found that Fsk had no effect on the C/N ratios of the ePKA-KTRs with the weakest and strongest bNLS scores (1.1 and 1.5) but triggered large and significant increases in the C/N scores of ePKA-KTR1.2/tdCherry, ePKA-KTR1.3/tdCherry, and ePKA-KTR1.4/tdCherry. Importantly, addition of leptomycin B, an inhibitor of nuclear protein export (40, 41), triggered a drop in the resting C/N ratios of ePKA-KTR1.2, 1.3, and 1.4 (Fig. 2*D*), indicating that their steady-state distribution involved active nuclear export, in addition to active nuclear import. In contrast, leptomycin B had no effect on the distribution of ePKA-KTR1.1, which is consistent with the hypothesis that the cytoplasmic accumulation of ePKA-KTR1.1 is mediated primarily by the passive barrier function of the nuclear pore. However, it should be noted that this barrier function is mediated primarily at the level of differential diffusion rates (37) and is not a binary switch between free diffusion and a complete absence of diffusion.

### KTR size impacts the dynamic range of ePKA-KTR1.2 and 1.4

Given that protein size appears to be critical to the design and testing of ePKA-KTRs, we next tested whether protein size might also affect the way that a well-designed eKTR actually performed. To explore this issue, we compared the Fsk responsiveness of eKTRs comprised of the ePKA-KTR1.2 and ePKA-KTR1.4 sensor domains fused to the N-terminus of either mCherry (∼34 kDa) or tdCherry (∼64 kDa) proteins. These four sensors were expressed in HEK293 cells, the cells were imaged by fluorescence microscopy before and after adding different concentrations of Fsk, and the resting and Fsk-induced C/N ratios were calculated. These experiments confirmed that all four of these ePKA-KTRs displayed a Fsk dose-dependent translocation to the cytoplasm (Fig. 2, *E* and *F*). Overall, the 64 kDa tdCherry forms of ePKA-KTR1.2 and ePKA-KTR1.4 both showed greater Fsk-induced accumulation in the cytoplasm than their 34 kDa mCherry forms, suggesting that increasing KTR size from 34 kDa to 64 kDa does indeed improve KTR performance characteristics. These results also indicate that the ePKA-KTR1.2/tdCherry sensor (NLS score = 10.5) responded particularly well at low concentrations of Fsk, whereas the C/N ratio of ePKA-KTR1.4/tdCherry (NLS score = 12.5) increased across the entire range of tested Fsk concentrations (Fig. 2, *E* and *F*), suggesting that varying bNLS strength is one way to generate ePKA-KTRs tuned to different levels of protein kinase activity.

### KTR oligomerization also affects KTR dynamics

We next tested whether small oligomerizing peptides might also affect KTR dynamics. Towards this end, we created eKTRs comprised of the ePKA-KTR1.2 sensor domain fused to the N-terminus of mScarlet-I (42) and mScarlet-I/BCR, which carries the BCR homo-oligomerization peptide at its C-terminus. The BCR peptide forms anti-parallel homodimers and homotetramers (43), and thus, we expect ePKA-KTR1.2/mScarlet-I/BCR to form oligomers of ∼70 to 140 kDa that have 2 to 4 copies of the kinase sensor domain. We transfected HEK293 cells with plasmids designed to express ePKA-KTR1.2/mScarlet-I and ePKA-KTR1.2/mScarlet-I/BCR, grew the cells for 2 days, and examined their subcellular distribution following a 30 min incubation with various concentrations of Fsk (30 uM). These experiments revealed that Fsk-induced increases in C/N ratio were higher for ePKA-KTR1.2/mScarlet-I/BCR than for ePKA-KTR1.2/mScarlet-I, especially at the lower range of Fsk concentrations (1 or 3 μM) (Fig. 1*G*). How much of this difference is attributable to differences in KTR size and how much to differences in kinase sensor domain copy number remains to be determined.

### Increasing NLS strength and oligomerization enhances ERK-KTR dynamic range

**Figure 3:**
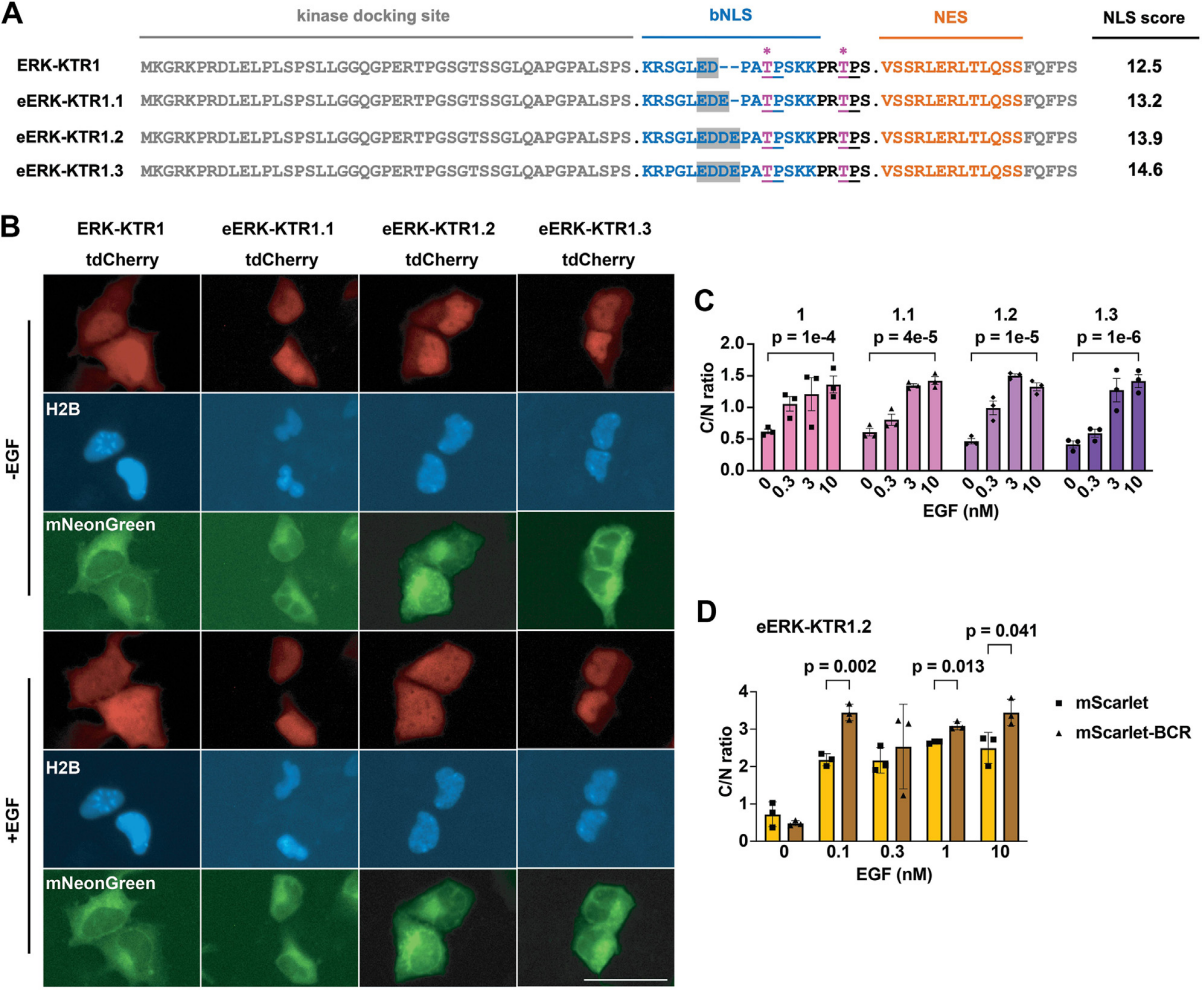
**Tuning the NLS of ERK-KTR.***A*, amino acid sequence alignments of ERK-KTR sensor domains, showing the kinase docking site, bNLS, (*pink*, *asterisk*) PKA phosphorylation sites, and NES, with predicted NLS strength scores listed to the *right*. Bar represents 50 μm. *B*, representative live-cell fluorescence images of HEK293 cells expressing H2B/mTagBFP2, mNeonGreen, and variants of ERK-KTR/tdCherry proteins. −EGF refers to fluorescence images taken prior to the addition of EGF, while +EGF refers to fluorescence micrographs taken 15 min after the addition of 10 nM EGF. Images are from the same cells observed prior to and after addition of EGF. *C*, bar graph showing C/N ratios of eERK-KTR1.1, 1.2, and 1.3/tdCherry1, following 15 min incubation in 0, 0.3, 3, or 10 nM EGF. *D*, C/N ratios of eERK-KTR1.2/mScarlet (*gold*) or eERK-KTR1.2/mScarlet/BCR (*brown*), after 15 min incubation in 0, 0.1, 0.3, 1, or 10 μM Fsk. The data for these bar graphs came from the analysis of independent groups of cells. All data in this figure are from a minimum of three independent biological replicates, the data for all bar graphs came from the analysis of independent groups of cells, the error bars reflect the SEM, and *p* values were calculated by two-way ANOVA.

Regot *et al.* also developed a KTR for measuring ERK activity (27), which we refer to as ERK-KTR1. Multiple studies have verified that ERK-KTR1 is an extremely useful tool for monitoring ERK activity (11, 29, 30, 44), and consistent with its obvious utility, the cNLS Mapper (39) score of its bNLS is high, 12.5 (Fig. 3*A*). Even so, we used cNLS Mapper (39) to develop variants of the ERK-KTR1 kinase sensor domain with higher predicted NLS strengths, which we refer to as eERK-KTR1.1, 1.2, and 1.3 (Fig. 3*A*). We generated plasmids designed to express these sensor domains at the N-terminus of tdCherry, transfected these into HEK293 cells, exposed the cells to EGF (10 nM) for 15 min, and used fluorescence microscopy to measure the subcellular distribution and C/N ratio of these large (64 kDa) fluorescent KTRs. All four of these ERK KTRs display an EGF dose-dependent translocation to the cytoplasm and a significant increase in their C/N ratio (Fig. 3, *A* and *B*). As expected, increasing the strength of the bNLS generated eERK-KTRs with a lower resting C/N ratio and a broader dynamic range, although the magnitude of these differences was not large.

To determine whether KTR oligomerization, size, and/or sensor domain copy number might affect ERK KTR dynamics, we created plasmids designed to express either eERK-KTR1.2/mScarlet-I or eERK-KTR1.2/mScarlet-I/BCR. HEK293 cells were transfected with these plasmids, incubated for 2 days, exposed to vehicle or EGF (10 nM) for 15 min, and examined by fluorescence microscopy. Calculation of C/N ratios revealed that eERK-KTR1.2/mScarlet-I/BCR displayed a 6-fold dynamic range in response to 0.1 nM EGF but was unable to respond to changes at higher levels of EGF, whereas eERK-KTR1.2/mScarlet had a similar sensitivity but smaller dynamic range (Fig. 3*D*). These results support the idea that BCR-mediated oligomerization of small KTRs creates KTRs of unique sensitivity.

### Marking cytoplasm and nucleus with ER/mTagBFP2

In the course of these experiments, we observed that the H2B/mTagBFP2 fusion protein had an unexpected effect on cell size and cell division. To avoid these effects, we generated an endoplasmic reticulum (ER)-localized form of mTagBFP2 (ER/mTagBFP2) that carries a secretory signal sequence (45, 46) at its N-terminus and an ER retrieval signal (KDEL) at its C-terminus (47). This ER marker localizes to both the nuclear envelope and to ER tubules that extend throughout the cytoplasm, allowing us to definitively mark both the nucleus and the cytoplasm with this one fluorescent protein (Fig. S1*A*). Furthermore, when we measured the C/N ratio of ePKA-KTR1.2/tdCherry in cells co-expressing ER/mTagBFP2, H2B/mTagBFP2, or 3xNLS/mTagBFP2, we found that the deduced C/N ratio of ePKA-KTR1.2/tdCherry was highest in cells expressing ER/mTagBFP2 (Fig. S1*B*). These results raise the possibility that KTR dynamics might be affected by co-expressing NLS-containing proteins. All subsequent studies of PKA and ERK KTRs were performed in cells co-expressing ER/mTagBFP2.

### Responsiveness of ePKA-KTR1.2 to PKA agonists and inhibitors

**Figure 4:**
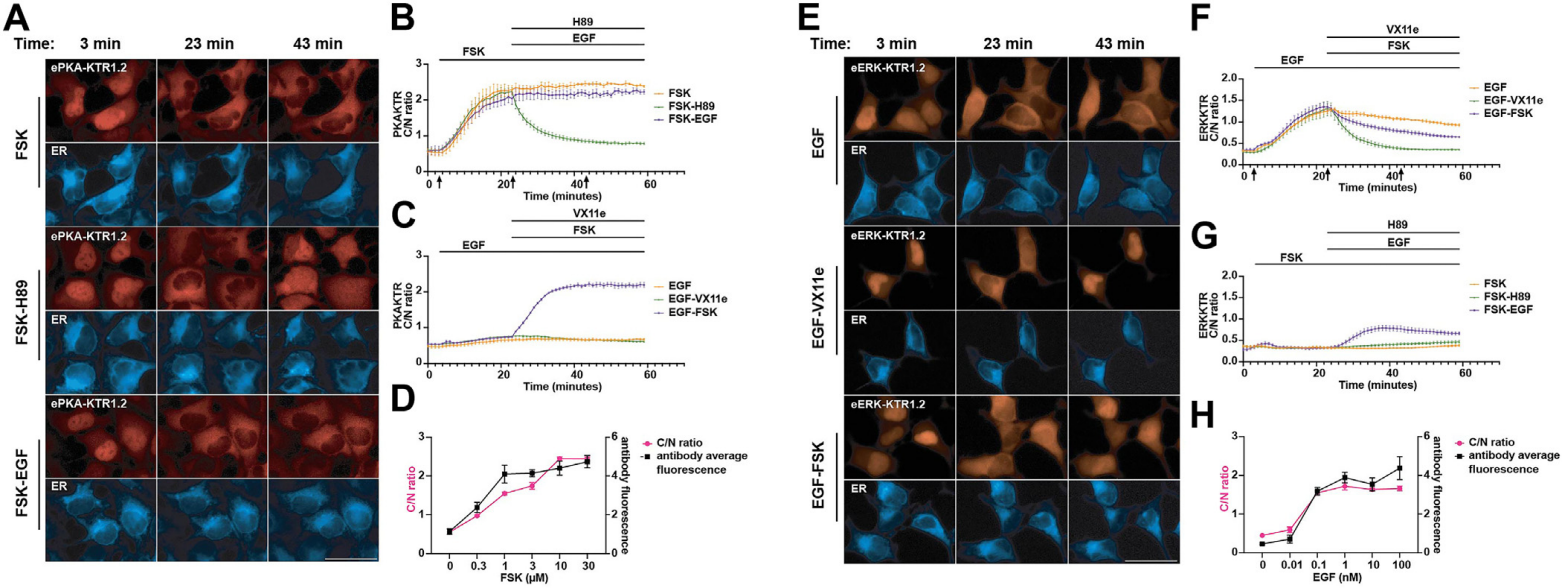
**Kinetics of ePKA-KTR1.2 and eERK-KTR1.2 responses.***A*, representative fluorescence micrographs of ePKA-KTR1.2/tdCherry-expressing, ER/mTagBFP2–expressing HEK293 cells at t = 3 min, t = 23 min, and t = 43 min, with cells exposed to Fsk alone at t = 3 min, Fsk at t = 3 min, and H89 at t = 23 min, or Fsk at t = 3 min and EGF at t = 23 min. Bar represents 50 μm. *B*, plot of ePKA-KTR1.2/tdCherry C/N ratios at every minute in cells exposed to (*orange*) Fsk only, (*green*) Fsk followed by H89, and (*purple*) Fsk followed by EGF. Lines at *top* denote times of chemical addition; arrows denote the times at which images in (*A*) were taken. *C*, plot of ePKA-KTR1.2/tdCherry C/N ratios every minute, in cells exposed to (*orange*) EGF alone, (*green*) EGF then VX11e, or (*purple*) EGF then Fsk. *D*, double plot of (*red*) ePKA-KTR1.2/tdCherry C/N ratios and (*black*) anti-phosphoPKA antibody staining intensity. In these experiments, the cells were incubated for 10 min in 0, 0.3, 1, 3, 10, and 30 μM Fsk, after which the cells were fixed, permeabilized, processed for immunofluorescence microscopy, and imaged, with phospho-PKA levels and C/N ratios calculated from the digital image files. *E*, representative fluorescence micrographs of eERK-KTR1.2/emiRFP670-expressing HEK293 cells at t = 3 min, t = 23 min, and t = 43 min, with cells exposed to EGF alone at t = 3 min, EGF at t = 3 min and VX11e at t = 23 min, or EGF at t = 3 min and Fsk at t = 23 min. Bar represents 50 μm. *F*, plot of eERK-KTR1.2/emiRFP670 C/N ratios at every minute in cells exposed to (*orange*) EGF only, (*green*) EGF then VX11e, and (*purple*) EGF then Fsk. *G*, plot of eERK-KTR1.2/emiRFP670 C/N ratios at every minute in cells exposed to (*orange*) Fsk only, (*green*) Fsk then H89, and (*purple*) Fsk then EGF. *H*, double plot of (*red*) eERK-KTR1.2/emiRFP670 C/N ratios and (*black*) anti-phospho-ERK antibody staining intensity. In these experiments, the cells were incubated for 5 min in 0, 0.01, 0.1, 1, 10, or 100 nM EGF, after which the cells were fixed, permeabilized, processed for immunofluorescence microscopy, and imaged, with phospho-ERK levels and C/N ratios calculated from the digital image files. All data in this figure are from a minimum of three independent biological replicates, the data for all graphs came from the analysis of independent groups of cells, and the error bars reflect the SEM.

Given that the sensor domain of ePKA-KTR1.2 appeared to specify a strong nuclear localization in unstimulated cells and robust translocation to the cytoplasm in response to Fsk, we elected to interrogate its C/N ratio on a minute-by-minute timescale in response to known activators and inhibitors of PKA. Towards this end, we created a stable, transgenic HEK293 cell line that continuously expressed both ePKA-KTR1.2/tdTomato and ER/mTagBFP2 and then examined the subcellular distribution of ePKA-KTR1.2/tdTomato by live cell imaging on an EVOS M7000 microscope equipped with a temperature-controlled stage set to 37 °C. Visualization of ePKA-KTR1.2/tdTomato in these cells confirmed its resting nuclear localization in unstimulated HEK293 cells (Fig. 4*A*) (C/N ratio of ∼0.6). Addition of Fsk (3 μM) at t = 3 min induced the translocation of ePKA-KTR1.2/tdTomato to the cytoplasm, a change that reached significance within 4 min (1.5-fold increase, *p* = 0.04), was half-maximal at 8 min (2.7-fold increase, *p* = 0.04), appeared to be saturated by 20 min (4-fold increase, *p* = 0.0005), and remained relatively constant thereafter (Fig. 4*B*). In a parallel culture, exposing cells to Fsk (3 μM) at t = 3 min and then the PKA inhibitor H89 (50 μM) at t = 23 min resulted in a rapid drop in the ePKA-KTR1.2/tdTomato C/N ratio, a decline that reached significance within 1 min (0.8-fold, *p* = 0.005), was half-maximal by 3 min (0.7-fold, *p* = 0.0005), and appeared to be complete by t = 11 min (0.5-fold, *p* = 0.005) (movie S1).

Previous studies have established that EGF, acting through the EGF/EGFR/Ras/Raf/MEK/ERK signaling pathway can, under some conditions, activate PKA signaling (48, 49, 50, 51). Although adding EGF (10 nM at t = 23 min) had no apparent effect on the C/N ratio of ePKA-KTR1.2/tdTomato in cells previously stimulated with Fsk (Fig. 4*B*), adding EGF to otherwise unstimulated cells (10 nM, at t = 3 min) did trigger a slight and slow translocation of ePKA-KTR1.2/tdTomato towards the cytoplasm over the ensuing 20 min (Fig. 4*C*). This rise in its C/N ratio was reversed by subsequent addition of the ERK inhibitor VX11e (5 μM) at t = 23 min, indicating that the response was specific. Furthermore, when cells exposed to EGF at t = 3 min were subsequently exposed to Fsk (3 μM) at t = 23 min, the responsiveness of ePKA-KTR1.2/tTomato to Fsk was as or more rapid than we observed previously, reaching significance within 1 min (1.2-fold, *p* = 0.0012) and half-maximum at 5 min (1.9-fold, *p* = 0.001).

PKA regulatory subunit phosphorylation correlates with PKA activity, and as a result, immunostaining cells with anti-phospho-PKA antibodies has become a useful way measure of PKA activity in individual cells (52, 53, 54, 55). We exposed ePKA-KTR1.2/tdTomato-expressing cells to a range of Fsk concentrations for 10 min, at 37 °C, after which the cells were fixed, permeabilized, and stained with anti-phospho-PKA antibodies and used fluorescence microscopy to measure both the ePKA-KTR1.2/tdTomato C/N ratio and the intensity of anti-phospho-PKA staining. Digital image analysis was performed on at least 10 cells from three independent ROIs, from a minimum of three independent trials. The ePKA-KTR1.2/tdTomato C/N ratios and the anti-phospho-PKA immunostaining intensities were of similar sensitivities and dynamic range (Fig. 4*D*). These results should not be interpreted as evidence of equivalent response kinetics, but they do suggest a similar integrated response over the fixed time period of this experiment, providing further validation that ePKA-KTR1.2/tdTomato is a useful tool for measuring relative PKA activities.

### Kinetics, specificity, and sensitivity of eERK-KTR1.2

We also interrogated the response times of eERK-KTR1.2. Using an HEK293 cell line stably expressing eERK-KTR1.2/emiRFP670 (∼46 kDa in size) and ER/mTagBFP2, we found that EGF (10 nM, added at t = 3 min) induced a sharp increase in the eERK-KTR1.2/emiRFP670 C/N ratio that reached significance at 3 min (1.5-fold, *p* = 0.002), was half-maximal at 9 min (2.4-fold, *p* = 0.003), plateaued at 20 min (4-fold, *p* = 0.0015), and declined slowly thereafter (0.7-fold between t = 25 and t = 59, *p* = 0.007) (Fig. 4, *E* and *F*). In a parallel culture, subsequent addition of the ERK inhibitor VX11e (5 μM) at t = 23 min triggered a return of eERK-KTR1.2/emiRFP670 to the nucleus and a drop in its C/N ratio that reached significance by 8 min (0.5-fold, *p* = 0.049) and appeared complete by 15 min (0.3-fold, *p* = 0.04) (Fig. 4, *E* and *F*) (movie S2).

Previous studies have established that PKA strongly inhibits ERK signaling (56, 57, 58, 59, 60). As suggested by these prior studies, addition of Fsk at t = 23 min (3 μM) to cells activated previously with EGF (10 nm, added at t = 3 min) resulted in a slow decline in the eERK-KTR1.2 C/N ratio (Fig. 4, *E* and *F*; movie S3). Consistent with these results, adding Fsk first (3 μM at t = 3 min) resulted in a smaller EGF-induced rise in the eERK-KTR1.2/emiRFP670 C/N ratio (Fig. 4, *E* and *G*; movie S3). ERK also activates PKC (9, 61, 62), and thus, eERK-KTR1.2/emiRFP670 also translocated to the cytoplasm in response to the PKC agonist phorbol myristate acetate (PMA) (63) at 80 nM t = 3 min), shown here by a sharp rise in its C/N ratio, which subsequently declined in response to the ERK inhibitor VX11e (5 μM) (Fig. S2).

Like PKA, ERK phosphorylation correlates with ERK activity (64), and staining with anti-phospho-ERK antibodies is a standard way to measure ERK activity in individual cells. To determine whether the nucleocytoplasmic distribution of eERK-KTR1.2/emiRFP670 correlated with levels of anti-phospho-ERK immunostaining, we exposed the eERK-KTR1.2/emiRFP670-expressing cell line to various concentrations of EGF for 5 min, then fixed the cells, permeabilized them, and processed them for immunofluorescence microscopy using anti-phospho-ERK antibodies, taking care to also measure the C/N ratios of eERK-KTR1.2/emiRFP670 at each concentration. When plotted relative to one another, the two techniques appeared to correlate well with one another (Fig. 4*H*), suggesting that they display a similar integrated response to ERK activity over the fixed time period of this experiment. However, these similarities between eERK-KTR1.2/emiRFP670 C/N ratios and anti-phospho-ERK immunostaining intensity should not be interpreted as evidence of equivalent response kinetics.

### Co-expression of ePKA-KTRs and eERK-KTRs illuminates unbalanced crosstalk between the PKA and ERK pathways

**Figure 5:**
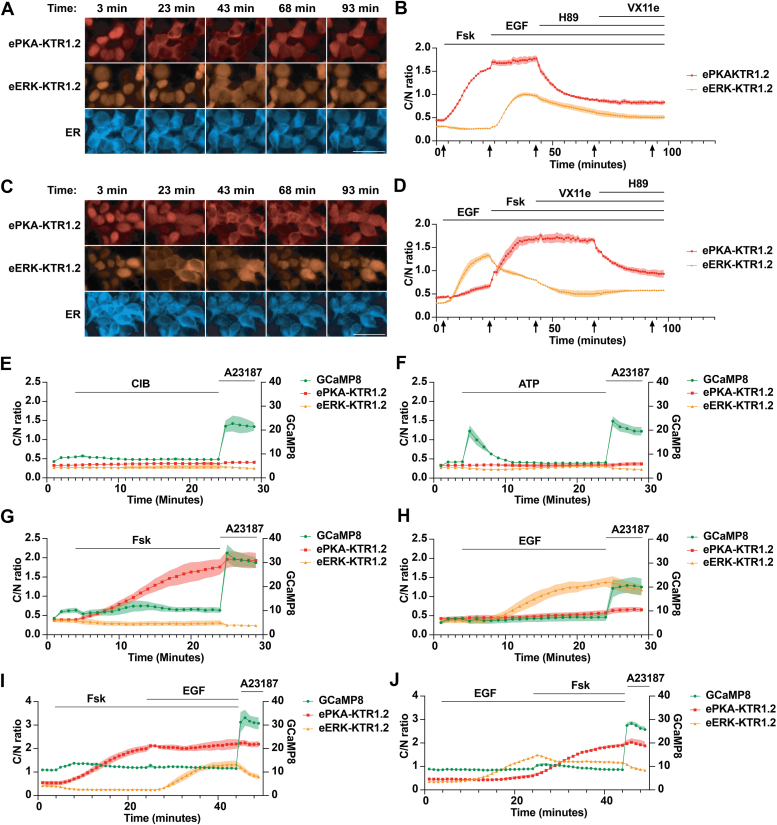
**Simultaneous measurement of PKA activity, ERK activity, and calcium.***A*, representative fluorescence images of HEK293 cells co-expressing ePKA-KTR1.2/tdTomato, eERK-KTR1.2/emiRFP670, and ER/mTagBFP2 exposed to Fsk at t = 3 min, then EGF at t = 23 min, then H89 at t = 50 min, then VX11e at t = 75 min, with images collected at t = 3, 23, 43, 68, and 93 min. Bar represents 50 μm. *B*, plot of C/N ratios of (*red*) ePKA-KTR1.2/tdTomato and (*orange*) eERK-KTR1.2/emiRFP670 every 60 s, showing their responses to Fsk, then EGF, then H89, then VX11e. Lines at the *top* denote the times of chemical addition, and arrows at the *bottom* denote the times at which the images of (A) were collected. *C*, representative fluorescence images of HEK293 cells co-expressing ePKA-KTR1.2/tdTomato, eERK-KTR1.2/emiRFP670, and ER/mTagBFP2 exposed to Fsk at t = 3 min, then EGF at t = 23 min, then H89 at t = 50 min, then VX11e at t = 75 min. Bar represents 50 μm. *D*, plot of C/N ratios of (*red*) ePKA-KTR1.2/tdTomato and (*orange*) eERK-KTR1.2/emiRFP670 every 60 s, showing their responses to EGF, then Fsk, then VX11e, then H89. *E*–*J*, plots of (*green*) GCaMP8 fluorescence intensity, (*red*) ePKA-KTR1.2/tdTomato C/N ratio, and (*dark red*) eERK-KTR1.2/emiRFP670 C/N ratio calculated from images collected at every minute as the cells were exposed to (*E*) CIB then A23187, (*F*) ATP then A23187, (*G*) Fsk then A23187, (*H*) EGF then A23187, (*I*) Fsk then EGF then A23187, or (*J*) EGF then Fsk then A23187. Lines at the *top* denote the times of chemical addition, C/N ratios are reflected in the *left* Y-axis, and GCaMP fluorescence intensity is reflected in the *right* Y-axis. All data in this figure are from a minimum of three independent biological replicates, the data for all graphs came from the analysis of independent groups of cells, and the error bars reflect the SEM.

To determine whether co-expression of ePKA-KTR1.2/tdTomato and eERK-KTR1.2/emiRFP670 could be used to monitor both pathways in individual live cells, we created an HEK293 cell line that stably co-expressed ePKA-KTR1.2/tdTomato, eERK-KTR1.2/emiRFP670, and ER/mTagBFP2. Addition of Fsk triggered the nuclear export ePKA-KTR1.2/tdTomato but caused the C/N ratio of eERK-KTR1.2/emiRFP670 to fall slightly, while subsequent addition of EGF led to an intermediate rise in the C/N ratio of eERK-KTR1.2/emiRFP670 C/N ratio and also appeared to increase the C/N ratio of ePKA-KTR1.2/tdTomato (Fig. 5, *A* and *B*) (movies S4A (ePKA-KTR1.2) and S4B (eERK-KTR1.2)). Inhibition of PKA with H89 led to a rapid drop in the C/N ratio of ePKA-KTR1.2/tdTomato and possibly enhanced the time-dependent decay in the C/N ratio of eERK-KTR1.2/emiRFP670. Adding VX-11e after these treatments had no apparent effect on either of these eKTRs.

We next added agonists in the complementary order of EGF, Fsk, VX11e, and H89 (Fig. 5, *C* and *D*) (movies S5A (ePKA-KTR1.2) and S5B (e*ERK-KTR1.2*)). As expected, EGF induced a sharp rise in the C/N ratio of eERK-KTR1.2/emiRFP670 and also increased the C/N ratio of ePKA-KTR1.2/tdTomato, though in these experiments, the EGF-induced activation of PKA signaling was of greater amplitude than we observed previously in Fig. 4. Subsequent addition of Fsk caused a steep rise in the C/N ratio of ePKA-KTR1.2/tdTomato, while at the same time inhibiting ERK, seen here by the drop in C/N ratio for eERK-KTR1.2/emiRFP670. Later addition of VX-11e seemed to drive the C/N ratio of eERK-KTR1.2/emiRFP670 lower but had no effect on that of ePKA-KTR1.2/tdTomato. Finally, addition of H89 triggered a sharp drop in the C/N ratio of ePKA-KTR1.2/tdTomato and a slight increase in the C/N ratio of eERK-KTR1.2/emiRFP670. In summary, co-expression of these eKTRs in the same cells allows the visualization of PKA and ERK activities in response to their direct agonists and inhibitors, as well as their unbalanced crosstalk with each other.

### Simultaneous live cell imaging of PKA activity, ERK activity, and calcium

To determine whether these eKTRs could be multiplexed with a calcium biosensor, we transfected the above cell line with a plasmid that expresses the genetically encoded calcium indicator GCaMP8s (65). Cells incubated in calcium imaging buffer (CIB) maintained low C/N ratios of both eKTRs and low GCaMP8s fluorescence, though addition of the calcium ionophore A23187 (200 μM) triggered an instantaneous rise in GCaMP8s fluorescence intensity (Fig. 5*E*). Exogenous ATP induces a P2Y receptor-mediated, store-operated intracellular calcium release (66), and adding ATP to the medium (10 μM) triggered a transient rise in GCaMP fluorescence that returned to baseline within 7 to 8 min and had no effect on the C/N ratio of either ePKA-KTR1.2/tdTomato or eERK-KTR1.2/emiRFP670 (Fig. 5*F*). Adding Fsk induced a rise in the C/N ratio of ePKA-KTR1.2/tdTomato and a slight increase in GCaMP8s fluorescence and also appeared to trigger a substantially higher levels of GCaMP8s fluorescence in response to A23187 (Fig. 5*G*). Adding EGF induced the expected strong increase in the C/N ratio of eERK-KTR1.2/emiRFP670, as well as the expected slight rise in the C/N ratio of ePKA-KTR1.2/tdTomato, but no sensitization of the GCaMP8s sensor to A23187 (Fig. 5*H*). Adding these agents in succession had their expected effects, with Fsk inducing the C/N ratio of ePKA-KTR1.2/tdTomato, depressing the C/N ratio of eERK-KTR1.2/emiRFP670, and attenuating the response of eERK-KTR1.2/emiRFP670 to subsequent addition of EGF (Fig. 5*I*), while treating cells first with EGF-induced nuclear export of eERK-KTR1.2/emiRFP670 and a slight rise in the C/N ratio of ePKA-KTR1.2/tdTomato, with subsequent addition of Fsk inducing the C/N ratio of ePKA-KTR1.2/tdTomato while decreasing the C/N ratio of eERK-KTR1.2/emiRFP670. Furthermore, all cells treated with Fsk displayed ∼1.5 brighter GCaMP8s fluorescence in response to A23187. Given that A23187 equilibrates calcium between the medium and cytoplasm, it seems unlikely that the effect of Fsk on GCaMP8s fluorescence reflects an Fsk-induced effect on calcium. In light of these observations, it is worth noting that the calcium sensor domain of GCaMP8s has a consensus phosphorylation site for PKA, raising the possibility that PKA-mediated phosphorylation of GCaMP8s might increase its calcium-induced fluorescence.

### Superior performance of ePKA-KTR1.4 and eERK-KTR1.2 in DRG neurons

**Figure 6:**
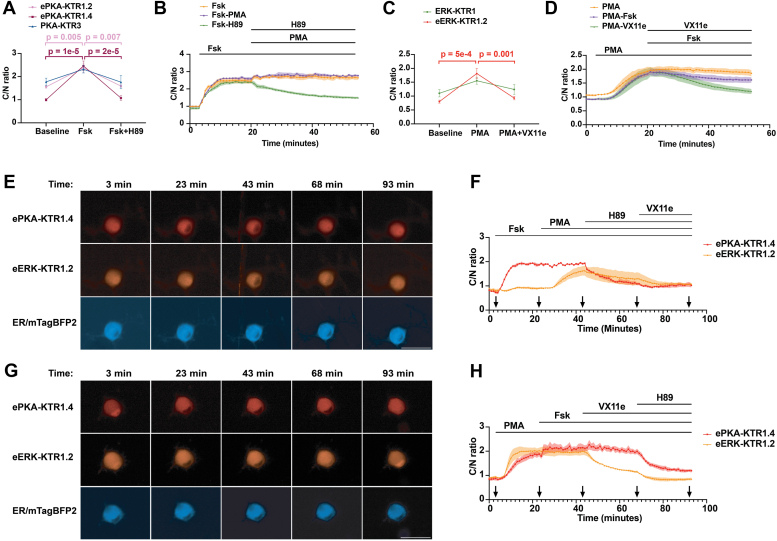
**Superior performance of ePKA-KTR1.4 and eERK-KTR1.2 in DRG neurons.***A*, graph of C/N ratios of (*pink*) ePKA-KTR1.2, (*purple*) ePKA-KTR1.4, and (*green*) PKA-KTR3 in DRG neurons, at baseline, in response to Fsk, and in response to subsequent addition of H89. The *p* values for the changes in PKA-KTR3 C/N ratio were 0.07 and 0.06, respectively. *B*, plot of C/N ratios of ePKA-KTR1.4/tdTomato collected every minute in response to (*orange*) Fsk alone, (*green*) Fsk followed later by the addition of H89, or (*purple*) Fsk followed later by the addition of PMA. Lines at the *top* denote the times of chemical addition. *C*, graph of C/N ratios of (*green*) ERK-KTR1 and (*orange*) eERK-KTR1.2 in DRG neurons at baseline, in response to PMA, and in response to subsequent addition of H89. The *p* values for the changes in ERK-KTR1 C/N ratio were 0.08 and 0.3, respectively. *D*, plot of C/N ratios of eERK-KTR1.2/emiRFP670 collected every minute in response to (*orange*) PMA alone, (*green*) PMA then VX11e, or (*purple*) PMA then VX11e. Lines at the *top* denote the times of chemical addition. *E*, representative fluorescence images of DRG co-expressing ePKA-KTR1.4/tdTomato, eERK-KTR1.2/emiRFP670, and ER/mTagBFP2, exposed sequentially to Fsk at t = 3 min, then PMA at t = 23 min, then H89 at t = 45 min, then VX11e at t = 70 min. Images were collected at t = 3, 23, 43, 68, and 93 min. Bar represents 50 μm. *F*, plot of C/N ratios of (*red*) ePKA-KTR1.4/tdTomato and (*orange*) eERK-KTR1.2/emiRFP670 at 1-min intervals, showing their responses to Fsk, then PMA, then H89, then VX11e. *G*, representative fluorescence images of DRG co-expressing ePKA-KTR1.4/tdTomato, eERK-KTR1.2/emiRFP670, and ER/mTagBFP2, exposed sequentially to PMA at t = 3 min, then Fsk at t = 23 min, then VX11e at t = 45 min, then H89 at t = 70 min. Images were collected at t = 3, 23, 43, 68, and 93 min. Bar represents 50 μm. *H*, plot of C/N ratios of (*red*) ePKA-KTR1.4/tdTomato and (*orange*) eERK-KTR1.2/emiRFP670 at 1-min intervals, showing their responses to PMA, then Fsk, then VX11e, then H89. All data in this figure are from a minimum of three independent biological replicates, the data for all bar graphs came from the analysis of independent groups of cells, the error bars reflect the SEM, and *p* values were calculated by two-way ANOVA.

Given our strong interest in nociceptive signaling, we next tested whether the eKTRs developed in HEK293 cells could be used to measure PKA and ERK signaling in primary sensory neurons of the dorsal root ganglion, which rely heavily on PKA and ERK signaling pathways (67, 68). Towards this goal, we surgically excised mouse dorsal root ganglia (DRG), dissociated their neurons, and plated them on coated chamber slides in the presence of a lentivirus designed to express ePKA-KTR1.2/tdTomato and ER/mTagBFP2. Three days later, the growth medium was replaced with CIB, the cells were incubated for 2 hours, and then the cells were imaged, also in CIB, at baseline, following exposure to Fsk (3 μM) for 15 min, and also after exposure to H89 (50 μM) for an additional 30 min, with images captured t = 0, t = 15 min, and t = 45 min (Fig. 6*A*). Calculation of the ePKA-KTR1.2/tdTomato C/N ratio revealed that this biosensor did not behave as expected in DRG neurons, as it displayed a baseline C/N ratio of ∼1.5, far greater than the C/N ratio it displayed in unstimulated HEK293 cells, which was ∼0.6. Furthermore, while the addition of Fsk did induce a significant increase in the ePKA-KTR1.2/tdTomato C/N ratio (*p* = 0.005), the dynamic range was shallow, as its C/N ratio only increased from ∼1.7 to ∼2.2. Subsequent addition of H89 returned its C/N ratio back to ∼1.7.

The fact that H89 did not reduce the C/N ratio of ePKA-KTR1.2/tdTomato below 1.7 suggested to us that the high resting C/N ratio of this biosensor reflects a DRG-specific difference in nucleocytoplasmic trafficking and is not caused by elevated PKA activity in these cells. We therefore transduced DRG neurons with a lentivirus designed to express ePKA-KTR1.4/tdTomato, which has a stronger bNLS (Fig. 1), and then repeated the preceding experiment. As hoped, the C/N ratio of ePKA-KTR1.4/tdTomato was substantially lower, ∼1, yet rose even higher than that of ePKA-KTR1.2/tdTomato in response to Fsk, displaying a broader dynamic range (∼1.3 C/N units), at greater statistical significance (*p* = 1 × 10^−5^), while also returning to baseline in response to H89 (Fig. 6*A*).

During the course of our studies, two additional PKA KTRs were reported, which we refer to as PKA-KTR2 (69) and PKA-KTR3 (31). To determine whether PKA-KTR2 or PKA-KTR3 displayed sensitivities similar, or better, than the ePKA-KTRs described earlier in this study, we fused their sensor domains to the N-terminus of mCherry and tdCherry, expressed them in HEK293 cells, and monitored their C/N ratio in response to Fsk. Neither PKA-KTR2/mCherry nor PKA-KTR2/tdCherry displayed a significant change in response to Fsk (Fig. S3, *A* and *B*), indicating that PKA-KTR2 sensor domain does not function in the context of these proteins. As for PKA-KTR3, we found that the C/N ratio of PKA-KTR3/mCherry rose significantly in response to Fsk, though it failed to rise above 1, resulting in a fairly shallow dynamic range (Fig. S3*C*). In contrast, the 64 kDa PKA-KTR3/tdCherry protein achieved a C/N ratio of >1.5 in response to Fsk (Fig. S3*D*), showing once again that increasing the size of KTRs above 40 kDa substantially improves their performance characteristics. In light of these encouraging results, we tested whether PKA-KTR3/tdCherry might be able to report on PKA activity in DRG neurons. Unfortunately, this was not the case, as PKA-KTR3/tdCherry had an extremely high resting C/N ratio in DRG neurons, ∼1.8, and failed to change significantly in response to Fsk, or to subsequent addition of H89 (*p* > 0.05) (Fig. 6*A*).

We followed these initial studies with a minute-by-minute analysis of ePKA-KTR1.4/tdTomato C/N ratios in transduced DRG neurons. In response to Fsk (3 μM, at t = 3 min), the C/N ratio of ePKA-KTR1.4/tdTomato rose rapidly, reaching saturation within 10 min and remaining high for the ensuing 40 min (Fig. 6*B*). In a parallel experiment, stimulation with Fsk (3 μM) at t = 3 min, followed by H89 (50 μM) at t = 20 min, resulted in the expected decline in ePKA-KTR1.4/tdTomato C/N ratio, though it did not return to baseline.

In light of these results, we next asked whether a stronger bNLS might also affect the responsiveness of ERK KTRs in isolated DRG neurons. Towards this end, DRG neurons were transduced with lentiviruses designed to express either ERK-KTR1/emiRFP670 or eERK-KTR1.2/emiRFP670, incubated for 3 days, and examined by fluorescence microscopy in the presence or absence of the PKC agonist PMA, a PKC agonist that also leads to ERK activation (our cultured DRG neurons did not respond to EGF). Adding PMA (80 nM) at t = 0 min had only a minor effect on the C/N ratio of ERK-KTR1/emiRFP670, increasing it from ∼1.1 to ∼1.4, and neither this increase nor the VX-11e-induced decrease was statistically significant (*p* > 0.05) (Fig. 6*C*). In contrast, PMA triggered a much larger increase in the C/N ratio of eERK-KTR1.2/emiRFP670, from ∼0.7 to ∼1.7, which was highly significant (*p* = 5 × 10^−4^). These results lend additional support to the hypothesis that detecting kinase activities in DRG neurons requires KTRs with a particularly strong bNLS. Furthermore, when we interrogated these responses on a minute-by-minute basis, we found that the PMA-induced rise in the C/N ratio of eERK-KTR1.2/emiRFP670 was inhibited strongly by VX-11e and weakly by Fsk (Fig. 6*D*).

To ensure that ePKA-KTR1.4/tdTomato and eERK-KTR1.2/emiRFP670 could be used to simultaneously monitor PKA and ERK activities in individual DRG neurons, we cotransduced DRG neurons with both lentiviruses, incubated them for 3 days, and then exposed them sequentially to Fsk, PMA, H89, and VX11e. Fsk induced the cytoplasmic translocation of ePKA-KTR1.4/tdTomato and also attenuated the PMA-induced increase in eERK-KTR1.2/emiRFP670 C/N ratio, while H89 induced a decline in the C/N ratio of both ePKA-KTR1.4/tdTomato and eERK-KTR1.2/miRFP670, and VX11e mediated the re-import of eERK-KTR1.2/emiRFP670 into the nucleus (Fig. 6, *E* and *F*). In a second set of experiments, sequential exposure to PMA, Fsk, VX11e, and H89 revealed PMA drove a rapid increase in the C/N ratio of both eERK-KTR1.2/emiRFP670 but also ePKA-KTR1.4/tdTomato, with the C/N ratio of ePKA-KTR1.4/tdTomato rising so high in response to PMA that subsequent addition of Fsk increased it only slightly (Fig. 6, *G* and *H*). Following these treatments, VX-11e caused a reduction in the C/N ratio of eERK-KTR1.2/emiRFP670, while later addition of H89 caused a decline in the C/N ratio of both KTRs. It should also be noted that the PMA-induced activation of PKA observed in DRG neurons was also observed in HEK293 cells expressing ePKA-KTR1.2/tdCherry (Fig. S4).

## Discussion

Among the integrative kinase biosensors, KTRs have the fastest response kinetics, reporting changes on a minute-by-minute timescale, similar to the kinetics of many kinase-regulated physiological processes. As such, KTRs fill an important temporal niche, recording kinase activities over periods of time that are ∼100-times faster than transcriptional reporters and conditionally stabilized reporters, which record kinase activities over hours-to-days (17, 18, 19, 20, 21, 22, 23, 24, 25, 26). Although the ability to monitor integrated kinase activities on a minute-by-minute basis is key to understanding the biological impacts of PKA, ERK, and other kinase signaling pathways, there has been a surprising lack of progress in improving the design and performance of KTRs, especially in comparison to the tremendous improvements that have been made for fast-response kinase biosensors (16, 65). In fact, the only notable improvement in KTRs over the past 10 years has been the R7A variant of ERK-KTR, which reduces cross-talk with a CDK1 inhibitor–sensitive pathway (30).

The data presented here show that KTR dynamic range and sensitivity can be improved considerably by (1) inhibiting the diffusion of KTRs through the nuclear pore by increasing their size and (2) ensuring that their bNLS is strong enough to direct their nuclear accumulation in unstimulated cells. Furthermore, our data suggest that there is value in developing a set of enhanced KTRs for each kinase, with the “set point” of each eKTR tuned, at least in part, by the strength of their bNLS. For example, we found that measurement of kinase activities in primary mouse DRG neurons appears to require eKTRs with a particularly strong bNLS sequence, namely ePKA-KTR1.4 and eERK-KTR1.2. It will be interesting to see whether these KTR design principles can be used to improve the dynamic range and sensitivities of other KTRs for other kinases, and also whether further improvements in KTR performance characteristics might be achieved by varying sensor domain copy number, KTR oligomerization, sensor domain position, and/or nature of the fluorescent protein.

The development of eKTRs linked to optically separable fluorescent proteins allowed us to follow the activation and inhibition of PKA and ERK, as well as changes calcium, when co-expressed with the calcium sensor jGCaMP8s in individual live cells. This has not previously been possible with a standard widefield fluorescence microscope. Co-expression of these biosensors also allowed us to verify the unbalanced nature of crosstalk between the PKA and ERK pathways, including PKA-mediated inhibition of ERK (56, 57, 58, 59, 60), EGF-mediated activation of PKA (48, 49, 50, 51), PMA/PKC-mediated activation of ERK (62), and PKA activation by PMA/PKC. Taken together, these results establish that ePKA-KTRs and eERK-KTRs are useful tools for monitoring the activation and inhibition of these key kinase signaling pathways in individual live cells, an ability that may be particularly revealing when applied to whole animals.

Our data also highlight the sensitivity of KTR C/N ratios to differences in experimental conditions. In addition to the obvious effect of low ambient temperature, which we found to slow KTR response times and required rigorous temperature controls in all reported experiments, KTR C/N ratios can also be affected by other experimental variations. For example, the cells used in all live cell imaging experiments were incubated for 2 h prior to the start of the experiment in CIB, which has no serum, and maintained in CIB through the duration of the experiment. In contrast, immunostained cells were grown continuously in Dulbecco’s modified Eagle’s medium (DMEM) containing 10% fetal bovine serum (FBS), and these changes in cell medium have the potential to affect C/N ratios. In addition, while live cell images were collected at the designated times, images of immunostained cells were collected only after the cells had been fixed, permeabilized, washed, and stained, a process known to suppress fluorescent protein fluorescence, especially of dim proteins like miRFP670[]. Furthermore, fixation alone can artifactually inflate KTR C/N ratios, as changes in a denominator affect an X/Y ratio more than equivalent changes in the numerator. These differences have the potential to explain why a 5 min EGF treatment induced a larger rise in the eERK-KTR1.2/miRFP670 C/N ratio in fixed, permeabilized, and immunostained cells (Fig. 4*H*) than in live cells (Fig. 4, *E* and *F*). In light of these considerations, we feel that comparisons of KTR C/N values generated under different experimental conditions should be avoided. In addition, it might also be useful to consider the relative merits of presenting KTR distributions by the mathematically justifiable formula of C/[C + N], which will vary from 0 to 1, as compared to the current convention of C/N, which varies dramatically by fluorescence intensity, tends towards infinity at low nuclear fluorescence.

## Experimental procedures

### Cell culture and DNA transfections

HEK293 cells and HEK293T cells (ATCC CRL-1573 and ATCC CRL-3216, respectively) were grown in complete medium (CM: DMEM (Thermo Fisher Scientific, 11965092) supplemented with 10% FBS (Gibco, 16000044) and 1% penicillin/streptomycin (Gibco, 15140122)). Both cell lines were obtained shortly before the start of this study, were interrogated visually to verify they retained their unique morphologies, and immunologically to confirm that they were of human origin (reactivity with antibodies specific to human antigens). Cultures were grown in 90% humidity and 5% CO_2_. Cells were transfected by lipofection using Lipofectamine 3000 according to the manufacturer’s instructions (Thermo Fisher Scientific, L3000015). Excised DRG neurons were cultured in neuron culture media, consisting of Neurobasal Plus (Gibco, A3582901) supplemented with B27 (Gibco, 17504044).

### Plasmids

Plasmids were assembled by the ligation of restriction enzyme–cleaved DNA fragments, which were derived from other plasmids or from PCR-amplified synthetic DNAs (geneblocks, IDT Inc). Plasmid architectures were confirmed by a combination of restriction enzyme mapping and DNA sequencing, with all sequences derived from amplified fragments confirmed in their entirety by DNA sequence analysis. All KTR vectors, mNeonGreen vectors, and mTagBFP2 vectors are based on Gould lab pC or pLenti vectors (70). Plasmid descriptions, including deduced amino acid sequences of key ORF, are presented in the supplemental information (Table S1). Addgene was the source of the plasmids psPAX2 from Didier Trono (Addgene# 12260) and VSV.G from Tannishtha Reya (Addgene# 14888).

### Live cell fluorescence microscopy

Live-cell fluorescence widefield microscopy was carried out using the EVOS M7000 Imaging System (Thermo Fisher Scientific, AMF7000) and 20× objective, equipped with an EVOS Onstage Incubator (Thermo Fisher Scientific, AMC2000) set to 37 °C. Prior to imaging, growth medium was replaced with Hepes-buffered physiological imaging media (10 mM Hepes, 130 mM NaCl, 3 mM KCl, 2.5 mM CaCl_2_, 0.6 mM MgCl_2_, NaHCO_3_, 10 mM glucose) for 2 hours prior to analysis. Images of HEK293 cells were captured by automated robotic imaging of four independent fields of view, while images of DRG neurons were captured from 10 independent fields of view.

### Immunofluorescence microscopy

HEK293 cells expressing eKTRs were grown in DMEM/10% FBS at 37 °C. Drugs were added to the DMEM/FBS culture medium at 37 °C and incubated for either 10 min (Fsk-stimulated cells) or 5 min (EGF-stimulated cells), then fixed by incubation in 4% formaldehyde in PBS for 30 min, and permeabilized with by incubation in 1% Triton X-100 in PBS for 5 min. Cells were then washed in PBS, incubated with primary antibodies for phospho-PKA (9101S, Cell Signaling Technology) and phospho-ERK (ab32390, Abcam). Specificity of these antibodies for phospho-PKA and phospho-ERK were established by the manufacturer and confirmed by us due to their low background in unstimulated cells and increasing immunoreactivity to cells exposed to the PKA agonist Fsk and the ERK agonist EGF, respectively. Following the incubation with these primary antibodies, cells were washed 3× with PBS and incubated with secondary Alexa Fluor 488–labeled donkey anti-rabbit antibodies (Jackson ImmunoResearch), which were validated by their lack of reactivity against of unlabeled cells. The resultant fixed, immunostained, KTR-expressing cells were imaged using the EVOS M7000 Imaging System (Thermo Fisher Scientific, AMF7000) equipped with a 20× objective. KTR C and N fluorescence levels were quantified by taking an averaged fluorescence intensity from a pair of cytoplasmic and nuclear ROIs and then calculating C/N ratios of the respective eKTRs, while at the same time calculating the AF488 fluorescence intensity of both ROIs of each cell and normalizing them to the value observed in control cells stained with secondary antibody only. In unstimulated cells, anti-phospho-PKA and anti-phospho-ERK immunostaining was similar to background.

### Image analysis and quantification of C/N ratios

Images were interrogated using NIS Elements software (RRID:SCR_014329) version 4.60. For endpoint multicolor image analyses, a pair of ROIs representing a randomly selected area in the nucleus and cytoplasm for each cell was drawn based on either a combination of H2B/mTagBFP2 and mNeonGreen fluorescence distributions in the experiments of Figs. 1 and 2 or ER/mTagBFP2 distribution alone in all subsequent experiments. For each image, a minimum of 10 cells were analyzed. KTR fluorescence intensity in the cytoplasmic and nuclear compartments was calculated from the average pixel fluorescence intensity within each compartment ROI, and the C/N fluorescence ratio was calculated for each cell, followed by averaging across all assayed cells in each image to create ROI data points, which were then treated as individual data points across a minimum of three biological replicates. Calculations of mean, SEM, and ANOVA *p* values were carried out using GraphPad Prism. Image analyses were conducted in a double-blinded fashion. For time course analyses, multicolor images were first registered in Fiji (ImageJ) and exported as movies. Analytical procedures were similar to those described above, including for the ROI/ratio analysis, the statistical analysis, and the minimum numbers of cells, biological replicates, and other variables. The positions of cytosolic and nuclear ROIs in each cell were checked between the first and last frame of the movies (using reference marker channels), to account for mispositioning over time due to cell movement over the course of the experiment. The Python script for KTR analysis is available upon request.

### Agonists, inhibitors, and antibodies

A23187 (Sigma, C7522), ATP (Sigma, A26209), Forskolin (Cayman Chemical, 11018), H89 (MedChemExpress, HY-15979A), EGF (Gibco, PHG0311L), VX-11e (Selleckchem, S7709), leptomycin B (Sigma Aldrich, L2913), and Phorbol 12-myristate 13-acetate (Sigma Aldrich, P1585) were obtained from commercial sources. Anti-phospho-ERK antibody (used at 1:1000; Cell Signaling Technology, 9101), anti-phospho-PKA antibody (used at 1:1000; Abcam, ab32390), and fluorophore-conjugated secondary anti-rabbit antibodies (used at 1:800; Jackson ImmunoResearch) were also obtained from commercial sources.

### Lentivirus production

HEK293T cells were seeded into 6-well plates the day prior to transfection in a volume of 2 mL CM per well. The next day (d0), the cells were at ∼90% confluency and transfected with equal amounts (by mole) of the pLenti transfer vector (designed to express the KTR-2a-ER/mTagBFP2 ORF), psPAX2, and VSV.G. The next day (d1), the 2ml culture medium was collected at place in a 50 mL sterile centrifuge tube, which was used as the collection vessel for all conditioned medium samples over the course of the lentivirus preparation. Cells from each well were released by trypsinization and re-seeded on a 10 cm plate with 15 mL CM. The next day (d2), 10 mL of supernatant was collected and added to the 2 mL of conditioned medium collected the previous day, and 10 mL of fresh cell culture media was added to each 10 cm dish of cells and the cells were incubated overnight. The next day (d3), 10 mL of supernatant was collected from each dish and added to the 12 mL of conditioned medium collected over the previous 2 days, and 10 mL of fresh cell culture media was added to each 10 cm dish of cells and the cells were again incubated overnight. The next day (d4), all 15 mL of conditioned medium was collected and added to the 22 mL of conditioned medium that had been collected from each sample over the previous 3 days, yielding a total volume of 37 mL conditioned medium from transfected cell population. The 37 mL was passed through a 450 nm pore size, syringe tip filter unit, yielding 37 mL of clarified conditioned medium. Lentivirus particles were pelleted from these samples by ultracentrifugation at 100,000*g* for 120 min (Optima L-90K, using a SW-32 Ti rotor, Beckman), and the pellets were resuspended in 1 mL of either CM or neuron culture media.

### Lentiviral transductions

For transduction, HEK293 cells were seeded into 24-well tissue culture plates at 10% confluency, grown overnight in CM, and then incubated with 50 to 100 μL of lentivirus preparation. Cells were then incubated for 2 to 4 more days prior to use in imaging or fluorescence activated cell sorting (FACS). For transduction of DRG neurons, 50 μl of lentivirus preparation was added to primary DRG neurons cultured in 300 μl of neuron culture medium. Live-cell imaging experiments were conducted 2 to 3 days after transduction.

### Fluorescence activated cell sorting

Lentivirus-transduced, KTR-expressing HEK293 cells were cultured for a minimum of 7 days post-transduction, after which they were trypsinized, together with the parental HEK293 cell line, and interrogated by flow cytometry using a Sony MA900 Cell Sorter. Flow cytometry confirmed that HEK293 cells displayed only background fluorescence, that some of the cells transduced with the ePKA-KTR1.2/tdTomato-2a-ER/mTagBFP2-expressing lentivirus displayed bright tdTomato and mTagBFP2 fluorescence, that some of the cells transduced with the eERK-KTR1.2/emiRFP670-2a-ER/mTagBFP2–expressing lentivirus displayed bright emiRFP670 and mTagBFP2 fluorescence, and that some of the cells co-transduced with both lentiviruses expressed bright tdTomato, emiRFP670, and mTagBFP2 fluorescence. We then used the Sony MA900 Cell Sorter to isolate subpopulations of these three fluorescent cell lines that displayed the highest 25% of fluorescence brightness, after which the three FACS-sorted cell lines were recovered in complete medium, expanded, and stocked.

### Mice, DRG isolation, culture, and transduction

All animal studies were approved by the Johns Hopkins University Animal Care and Use Committee (IACUC approval #MO23M18). C57BL/6 mice were maintained under standard conditions, with water and food provided ad libitum. To obtain DRG neurons, C57BL/6 mice of 4 to 6 weeks old were euthanized by CO_2_ inhalation followed by cervical dislocation prior to dissection. Under a dissection microscope, the spinal column was excised, bisected, and peeled to reveal dorsal root ganglia. Individual ganglia were picked using surgical forceps and placed in ice-cold PBS. Enzymatic digestion of connective tissue was carried out at 37 °C using Liberase TM (Roche, 5401119001) for 20 min, followed by Liberase TL (Roche, 5401020001) and papain enzyme suspension (Worthington, LS003126) for 20 min. Digestion was terminated by 2% bovine serum albumin (Sigma, A9418) in neuron culture media. Individual DRG neurons were released by repeated pipetting (20 times) of digested ganglia in 500 μl neuron culture media, which was performed up to 5 times, or until tissue dissociation was complete. Cells were pelleted at 300*g* for 3 min, resuspended in neuron culture media, and plated onto imaging chambers (Ibidi, 80807) pre-coated with 0.01% poly-D-lysine (Sigma Aldrich, P6407) and mouse laminin (Gibco, 23017015).

### Formal analysis

All data in this figure are from a minimum of three independent biological replicates, the data for all bar graphs came from the analysis of independent groups of cells, the error bars reflect the SEM, and *p* values were calculated by two-way ANOVA. Most analyses were blinded but some were not. Visual assessment of data points verified normality in the data, justifying our use of the parametric two-way ANOVA test.

## Data availability

All data are contained within the manuscript. Source data is available upon reasonable request, to the senior corresponding author, Dr Gould, at sgould@jhmi.edu. All materials are available upon request to the corresponding author, Dr Gould, at sgould@jhmi.edu, provided that a material transfer agreement is completed with Johns Hopkins University.

## Supporting information

This article contains supporting information.

## Conflicts of interest

S. J. T., M. J. C., and S. J. G. are co-inventors of proprietary materials described in this paper that are owned by Johns Hopkins University, and as a result, they may receive compensation related to their licensing and commercial use. All other authors declare that they have no conflicts of interest with the contents of this article.

## Supporting information

Movie S3

Movie S4A

Movie S4B

Movie S5A

Movie S5B
