# Supplementary material for: Enhanced kinase translocation reporters for simultaneous real-time measurement of PKA, ERK, and calcium
Source: J Biol Chem. 2025 Jan 13;301(3):108183. doi: 10.1016/j.jbc.2025.108183 (PMC11871455; doi:10.1016/j.jbc.2025.108183)
Supplement: Supporting Information [file mmc1.docx]

**SUPPORTING INFORMATION**

***Figure S1. ER/mTagBFP2 is a useful marker of both nucleus and cytoplasm***. (**A**) Fluorescence micrographs of HEK293 cells co-expressing ER/mTagBFP2 and ePKA-KTR1.2/tdCherry. The distribution of ER/mTagBFP2 determines the positioning of regions-of-interest’ (ROIs; white boxes) that are then used to count the relative fluorescence intensity of KTR proteins in the nucleus and cytoplasm, with the C/N ratio determined from the average fluorescence brightness of all pixels within each counting area. (**B)** C/N ratios of ePKA-KTR1.2 in HEK293 cells co-expressing (blue bars) the ER/mTagBFP2 marker (ER), (purple bars) the 3xNLS/mTagBFP2 marker (3xNLS), or (green bars) the H2B/mTagBFP2 marker (H2B). Cells were exposed to 0, 1, 3, 10, or 30 uM Fsk for 30 minutes, imaged by fluorescence microscopy, and C/N ratios were calculated from digital images. All data in this figure are from a minimum of three independent biological replicates, the data for all bar graphs came from the analysis of independent groups of cells, the error bars reflect the standard error of the mean, and *p* values were calculated by two-way ANOVA. ANOVA *p* values are denoted by * <0.05, and ** <0.01.

***Figure S2. PMA induces the nuclear export of eERK-KTR1.2/emiRFP670***. HEK293 cells expressing eERK-KTR1.2/emiRFP670 were imaged every 60 sec (orange) in response to PMA alone or (green) in response to PMA, followed 20 min later by addition of VX11e. All data in this figure are from a minimum of three independent biological replicates, the data for all bar graphs came from the analysis of independent groups of cells, and the error bars reflect the standard error of the mean.

***Figure S3. Increasing KTR size increases the dynamic range of PKA-KTR3***. HEK293 cells were transfected with plasmid vectors designed to express fusion proteins comprised of the sensor domains of PKA-KTR2(1) or PKA-KTR3(2) appended to the N-terminus of either mCherry, tdCherry. Two days later, cells were imaged by fluorescence microscopy prior to or after a 30 min. incubation in 30 mM Fsk, followed by calculation of C/N ratios in 10 cells in independent regions of interest (ROIs) from three biological replicates, shown here in bar graph form. (**A, B**) Both PKA-KTR2/mCherry, a ~34 kDa proteins, and PKA-KTR2/tdCherry, a ~64 kDa protein, had resting C/N ratios <1, and neither showed a Fsk-induced translocation to the cytoplasm. (**C**) PKA-KTR3/mCherry, a ~34 kDa protein, displayed a low resting C/N ratio and moved to the cytoplasm in response to Fsk, confirming that the PKA-KTR3 sensor domain is a useful reporter of PKA activity. However, Fsk-induced C/N ratio of PKA-KTR3/mCherry remained <1, and its dynamic range was relatively narrow, only ~2-fold. (**D**) In contrast, we found that PKA-KTR3/tdCherry, ~64 kDa, had a broader dynamic range, ~3-fold, showing once again that increasing KTR size can be sufficient to improve KTR performance characteristics. All data in this figure are from a minimum of three independent biological replicates, the data for all bar graphs came from the analysis of independent groups of cells, the error bars reflect the standard error of the mean, and *p* values were calculated by two-tailed t-test.

***Figure S4. PMA induces a mild activation of PKA***. Plot of C/N ratios for ePKA-KTR1.2/tdTomato at every minute in HEK293 cells exposed to (orange) PMA at t = 3 min, followed by the addition of the PKA inhibitor H89 at t = 23, or (green) PMA and H89 at t = 3, followed by addition of H89 again at t = 23 min. All data in this figure are from a minimum of three independent biological replicates, the data for all bar graphs came from the analysis of independent groups of cells, and the error bars reflect the standard error of the mean.

**MOVIE LEGENDS**

*Movie S1. Dynamics of ePKA-KTR1.2/tTomato localization in cells expressing ePKA-KTR1.2/tdTomato and ER-mTagBFP2*. Images were collected every minute, Fsk was added at t = 3 min, and H89 was added at t = 23 min.

Movie S2. *Dynamics of eERK-KTR1.2/emiRFP670 localization in cells expressing eERK-KTR1.2/emiRFP670 and ER-mTagBFP2*. Images were collected every minute, EGF was added at t = 3 min, and VX11e was added at t = 23 min.

Movie S3. *Dynamics of eERK-KTR1.2/emiRFP670 localization in cells expressing eERK-KTR1.2/emiRFP670 and ER-mTagBFP2*. Images were collected every minute, EGF was added at t = 3 min, and Fsk was added at t = 23 min.

Movie S4A. *Dynamics of ePKA-KTR1.2/tTomato localization in cells expressing ePKA-KTR1.2/tdTomato, eERK-KTR1.2/emiRFP670, and ER-mTagBFP2*. Images were collected every minute, Fsk was added at t = 3 min, EGF was added at t = 23 min, H89 was added at t = 43, and VX11e was added at 68 min.

Movie S4B. *Dynamics of eERK-KTR1.2/emiRFP670 localization in cells expressing ePKA-KTR1.2/tdTomato, eERK-KTR1.2/emiRFP670, and ER-mTagBFP2*. Images were collected every minute, Fsk was added at t = 3 min, EGF was added at t = 23 min, H89 was added at t = 43, and VX11e was added at 68 min.

Movie S5A. *Dynamics of ePKA-KTR1.2/tTomato localization in cells expressing ePKA-KTR1.2/tdTomato, eERK-KTR1.2/emiRFP670, and ER-mTagBFP2*. Images were collected every minute, EGF was added at t = 3 min, Fsk was added at t = 23 min, VX11e was added at t = 43, and H89 was added at 68 min.

Movie S5B. *Dynamics of eERK-KTR1.2/emiRFP670 localization in cells expressing ePKA-KTR1.2/tdTomato, eERK-KTR1.2/emiRFP670, and ER-mTagBFP2*. Images were collected every minute, EGF was added at t = 3 min, Fsk was added at t = 23 min, VX11e was added at t = 43, and H89 was added at 68 min.

***Table S1. Description, amino acid sequences, vector type, and plasmid number***. Vectors pC and pLenti were described previously(3). Amino acid sequences are represented in single letter code, with bold, black lettering for kinase sensor domains, red lettering for red and infrared fluorescent proteins, blue lettering for mTagBFP2, and black, unbolded lettering for other sequence elements (i.e. linker sequences, histone H2B sequence, C-terminal extensions, signal sequence, ER retrieval signal, and 3xNLS). The notation **|<….>|** denotes the position in the viral 2a peptide where the ribosome fails to make the peptide bond (between the upstream glycine and the downstream proline), resulting in the release of the upstream red or infrared fluorescent protein and the continued translation of the downstream blue fluorescent protein.

| **ORF Description** | **Deduced Amino Acid Sequences of the ORF** | **vector** | **plasmid #** |
| --- | --- | --- | --- |
| **PKA-KTR1/tdCherry.2a.H2B/mTagBFP2** | **MEMPEEPANSGHSLPPVYIYSPEYVSICDSLVKVPKRASMVNEDEAPSRRKASGQVSSRLERLTLQSS**GAPVSKGEEDNMAIIKEFMRFKVHMEGSVNGHEFEIEGEGEGRPYEGTQTAKLKVTKGGPLPFAWDILSPQFMYGSKAYVKHPADIPDYLKLSFPEGFKWERVMNFEDGGVVTVTQDSSLQDGEFIYKVKLRGTNFPSDGPVMQKKTMGWEASSERMYPEDGALKGEIKQRLKLKDGGHYDAEVKTTYKAKKPVQLPGAYNVNIKLDITSHNEDYTIVEQYERAEGRHSTGGMDELYKARGSAGSATTMVSKGEEDNMAIIKEFMRFKVHMEGSVNGHEFEIEGEGEGRPYEGTQTAKLKVTKGGPLPFAWDILSPQFMYGSKAYVKHPADIPDYLKLSFPEGFKWERVMNFEDGGVVTVTQDSSLQDGEFIYKVKLRGTNFPSDGPVMQKKTMGWEASSERMYPEDGALKGEIKQRLKLKDGGHYDAEVKTTYKAKKPVQLPGAYNVNIKLDITSHNEDYTIVEQYERAEGRHSTGGMDELYKASGSGATNFSLLKQAGDVEENPG**\|<....>\|**PSPAMPEPAKSAPAPKKGSKKAVTKAQKKGGKKRKRSRKESYSIYVYKVLKQVHPDTGISSKAMGIMNSFVNDIFERIAGEASRLAHYNKRSTITSREIQTAVRLLLPGELAKHAVSEGTKAITKYTSAKDPGGGSSRVSKGEELIKENMHMKLYMEGTVDNHHFKCTSEGEGKPYEGTQTMRIKVVEGGPLPFAFDILATSFLYGSKTFINHTQGIPDFFKQSFPEGFTWERVTTYEDGGVLTATQDTSLQDGCLIYNVKIRGVNFTSNGPVMQKKTLGWEAFTETLYPADGGLEGRNDMALKLVGGSHLIANAKTTYRSKKPAKNLKMPGVYYVDYRLERIKEANNETYVEQHEVAVARYCDLPSKLGHKLNGMDELYKGRSPGLNGGSGATNFSLLKQAGDVEENPG | **pC** | **S740** |
| **PKA-KTR1/mCherry.2a.H2B/mTagBFP2** | **MEMPEEPANSGHSLPPVYIYSPEYVSICDSLVKVPKRASMVNEDEAPSRRKASGQVSSRLERLTLQSS**GAPVSKGEEDNMAIIKEFMRFKVHMEGSVNGHEFEIEGEGEGRPYEGTQTAKLKVTKGGPLPFAWDILSPQFMYGSKAYVKHPADIPDYLKLSFPEGFKWERVMNFEDGGVVTVTQDSSLQDGEFIYKVKLRGTNFPSDGPVMQKKTMGWEASSERMYPEDGALKGEIKQRLKLKDGGHYDAEVKTTYKAKKPVQLPGAYNVNIKLDITSHNEDYTIVEQYERAEGRHSTGGMDELYKASGSGATNFSLLKQAGDVEENPG**\|<....>\|**PSPAMPEPAKSAPAPKKGSKKAVTKAQKKGGKKRKRSRKESYSIYVYKVLKQVHPDTGISSKAMGIMNSFVNDIFERIAGEASRLAHYNKRSTITSREIQTAVRLLLPGELAKHAVSEGTKAITKYTSAKDPGGGSSRVSKGEELIKENMHMKLYMEGTVDNHHFKCTSEGEGKPYEGTQTMRIKVVEGGPLPFAFDILATSFLYGSKTFINHTQGIPDFFKQSFPEGFTWERVTTYEDGGVLTATQDTSLQDGCLIYNVKIRGVNFTSNGPVMQKKTLGWEAFTETLYPADGGLEGRNDMALKLVGGSHLIANAKTTYRSKKPAKNLKMPGVYYVDYRLERIKEANNETYVEQHEVAVARYCDLPSKLGHKLNGMDELYKGRSPGLNGGSGATNFSLLKQAGDVEENPG | **pC** | **S739** |
| **ePKA-KTR1.1/tdCherry.2a.H2B/mTagBFP2** | **MEMPEEPANSGHSLPPVYIYSPEYVSICDSLVKVPKRASMVNEDEAPSKATRRASLGVSSRLERLTLQSS**GAPVSKGEEDNMAIIKEFMRFKVHMEGSVNGHEFEIEGEGEGRPYEGTQTAKLKVTKGGPLPFAWDILSPQFMYGSKAYVKHPADIPDYLKLSFPEGFKWERVMNFEDGGVVTVTQDSSLQDGEFIYKVKLRGTNFPSDGPVMQKKTMGWEASSERMYPEDGALKGEIKQRLKLKDGGHYDAEVKTTYKAKKPVQLPGAYNVNIKLDITSHNEDYTIVEQYERAEGRHSTGGMDELYKARGSAGSATTMVSKGEEDNMAIIKEFMRFKVHMEGSVNGHEFEIEGEGEGRPYEGTQTAKLKVTKGGPLPFAWDILSPQFMYGSKAYVKHPADIPDYLKLSFPEGFKWERVMNFEDGGVVTVTQDSSLQDGEFIYKVKLRGTNFPSDGPVMQKKTMGWEASSERMYPEDGALKGEIKQRLKLKDGGHYDAEVKTTYKAKKPVQLPGAYNVNIKLDITSHNEDYTIVEQYERAEGRHSTGGMDELYKASGSGATNFSLLKQAGDVEENPG**\|<....>\|**PSPAMPEPAKSAPAPKKGSKKAVTKAQKKGGKKRKRSRKESYSIYVYKVLKQVHPDTGISSKAMGIMNSFVNDIFERIAGEASRLAHYNKRSTITSREIQTAVRLLLPGELAKHAVSEGTKAITKYTSAKDPGGGSSRVSKGEELIKENMHMKLYMEGTVDNHHFKCTSEGEGKPYEGTQTMRIKVVEGGPLPFAFDILATSFLYGSKTFINHTQGIPDFFKQSFPEGFTWERVTTYEDGGVLTATQDTSLQDGCLIYNVKIRGVNFTSNGPVMQKKTLGWEAFTETLYPADGGLEGRNDMALKLVGGSHLIANAKTTYRSKKPAKNLKMPGVYYVDYRLERIKEANNETYVEQHEVAVARYCDLPSKLGHKLNGMDELYKGRSPGLNGGSGATNFSLLKQAGDVEENPG | **pC** | **S741** |
| **ePKA-KTR1.2/tdCherry.2a.H2B/mTagBFP2** | **MEMPEEPANSGHSLPPVYIYSPEYVSICDSLVKVPKRASMVNEDEAPSKRRASLGVSSRLERLTLQSS**GAPVSKGEEDNMAIIKEFMRFKVHMEGSVNGHEFEIEGEGEGRPYEGTQTAKLKVTKGGPLPFAWDILSPQFMYGSKAYVKHPADIPDYLKLSFPEGFKWERVMNFEDGGVVTVTQDSSLQDGEFIYKVKLRGTNFPSDGPVMQKKTMGWEASSERMYPEDGALKGEIKQRLKLKDGGHYDAEVKTTYKAKKPVQLPGAYNVNIKLDITSHNEDYTIVEQYERAEGRHSTGGMDELYKARGSAGSATTMVSKGEEDNMAIIKEFMRFKVHMEGSVNGHEFEIEGEGEGRPYEGTQTAKLKVTKGGPLPFAWDILSPQFMYGSKAYVKHPADIPDYLKLSFPEGFKWERVMNFEDGGVVTVTQDSSLQDGEFIYKVKLRGTNFPSDGPVMQKKTMGWEASSERMYPEDGALKGEIKQRLKLKDGGHYDAEVKTTYKAKKPVQLPGAYNVNIKLDITSHNEDYTIVEQYERAEGRHSTGGMDELYKASGSGATNFSLLKQAGDVEENPG**\|<....>\|**PSPAMPEPAKSAPAPKKGSKKAVTKAQKKGGKKRKRSRKESYSIYVYKVLKQVHPDTGISSKAMGIMNSFVNDIFERIAGEASRLAHYNKRSTITSREIQTAVRLLLPGELAKHAVSEGTKAITKYTSAKDPGGGSSRVSKGEELIKENMHMKLYMEGTVDNHHFKCTSEGEGKPYEGTQTMRIKVVEGGPLPFAFDILATSFLYGSKTFINHTQGIPDFFKQSFPEGFTWERVTTYEDGGVLTATQDTSLQDGCLIYNVKIRGVNFTSNGPVMQKKTLGWEAFTETLYPADGGLEGRNDMALKLVGGSHLIANAKTTYRSKKPAKNLKMPGVYYVDYRLERIKEANNETYVEQHEVAVARYCDLPSKLGHKLNGMDELYKGRSPGLNGGSGATNFSLLKQAGDVEENPG | **pC** | **S467** |
| **ePKA-KTR1.3/tdCherry.2a.H2B/mTagBFP2** | **MEMPEEPANSGHSLPPVYIYSPEYVSICDSLVKVPKRASMVNEDEAPAKRRASLGVSSRLERLTLQSS**GAPVSKGEEDNMAIIKEFMRFKVHMEGSVNGHEFEIEGEGEGRPYEGTQTAKLKVTKGGPLPFAWDILSPQFMYGSKAYVKHPADIPDYLKLSFPEGFKWERVMNFEDGGVVTVTQDSSLQDGEFIYKVKLRGTNFPSDGPVMQKKTMGWEASSERMYPEDGALKGEIKQRLKLKDGGHYDAEVKTTYKAKKPVQLPGAYNVNIKLDITSHNEDYTIVEQYERAEGRHSTGGMDELYKARGSAGSATTMVSKGEEDNMAIIKEFMRFKVHMEGSVNGHEFEIEGEGEGRPYEGTQTAKLKVTKGGPLPFAWDILSPQFMYGSKAYVKHPADIPDYLKLSFPEGFKWERVMNFEDGGVVTVTQDSSLQDGEFIYKVKLRGTNFPSDGPVMQKKTMGWEASSERMYPEDGALKGEIKQRLKLKDGGHYDAEVKTTYKAKKPVQLPGAYNVNIKLDITSHNEDYTIVEQYERAEGRHSTGGMDELYKASGSGATNFSLLKQAGDVEENPG**\|<....>\|**PSPAMPEPAKSAPAPKKGSKKAVTKAQKKGGKKRKRSRKESYSIYVYKVLKQVHPDTGISSKAMGIMNSFVNDIFERIAGEASRLAHYNKRSTITSREIQTAVRLLLPGELAKHAVSEGTKAITKYTSAKDPGGGSSRVSKGEELIKENMHMKLYMEGTVDNHHFKCTSEGEGKPYEGTQTMRIKVVEGGPLPFAFDILATSFLYGSKTFINHTQGIPDFFKQSFPEGFTWERVTTYEDGGVLTATQDTSLQDGCLIYNVKIRGVNFTSNGPVMQKKTLGWEAFTETLYPADGGLEGRNDMALKLVGGSHLIANAKTTYRSKKPAKNLKMPGVYYVDYRLERIKEANNETYVEQHEVAVARYCDLPSKLGHKLNGMDELYKGRSPGLNGGSGATNFSLLKQAGDVEENPG | **pC** | **S514** |
| **ePKA-KTR1.4/tdCherry.2a.H2B/mTagBFP2** | **MEMPEEPANSGHSLPPVYIYSPEYVSICDSLVKVPKRASMVNEEDEAPAKRRASLGVSSRLERLTLQSS**GAPVSKGEEDNMAIIKEFMRFKVHMEGSVNGHEFEIEGEGEGRPYEGTQTAKLKVTKGGPLPFAWDILSPQFMYGSKAYVKHPADIPDYLKLSFPEGFKWERVMNFEDGGVVTVTQDSSLQDGEFIYKVKLRGTNFPSDGPVMQKKTMGWEASSERMYPEDGALKGEIKQRLKLKDGGHYDAEVKTTYKAKKPVQLPGAYNVNIKLDITSHNEDYTIVEQYERAEGRHSTGGMDELYKARGSAGSATTMVSKGEEDNMAIIKEFMRFKVHMEGSVNGHEFEIEGEGEGRPYEGTQTAKLKVTKGGPLPFAWDILSPQFMYGSKAYVKHPADIPDYLKLSFPEGFKWERVMNFEDGGVVTVTQDSSLQDGEFIYKVKLRGTNFPSDGPVMQKKTMGWEASSERMYPEDGALKGEIKQRLKLKDGGHYDAEVKTTYKAKKPVQLPGAYNVNIKLDITSHNEDYTIVEQYERAEGRHSTGGMDELYKASGSGATNFSLLKQAGDVEENPG**\|<....>\|**PSPAMPEPAKSAPAPKKGSKKAVTKAQKKGGKKRKRSRKESYSIYVYKVLKQVHPDTGISSKAMGIMNSFVNDIFERIAGEASRLAHYNKRSTITSREIQTAVRLLLPGELAKHAVSEGTKAITKYTSAKDPGGGSSRVSKGEELIKENMHMKLYMEGTVDNHHFKCTSEGEGKPYEGTQTMRIKVVEGGPLPFAFDILATSFLYGSKTFINHTQGIPDFFKQSFPEGFTWERVTTYEDGGVLTATQDTSLQDGCLIYNVKIRGVNFTSNGPVMQKKTLGWEAFTETLYPADGGLEGRNDMALKLVGGSHLIANAKTTYRSKKPAKNLKMPGVYYVDYRLERIKEANNETYVEQHEVAVARYCDLPSKLGHKLNGMDELYKGRSPGLNGGSGATNFSLLKQAGDVEENPG | **pC** | **S515** |
| **ePKA-KTR1.5/tdCherry.2a.H2B/mTagBFP2** | **MEMPEEPANSGHSLPPVYIYSPEYVSICDSLVKVPKRASMVNEDEAPSKKRRASLGVSSRLERLTLQSS**GAPVSKGEEDNMAIIKEFMRFKVHMEGSVNGHEFEIEGEGEGRPYEGTQTAKLKVTKGGPLPFAWDILSPQFMYGSKAYVKHPADIPDYLKLSFPEGFKWERVMNFEDGGVVTVTQDSSLQDGEFIYKVKLRGTNFPSDGPVMQKKTMGWEASSERMYPEDGALKGEIKQRLKLKDGGHYDAEVKTTYKAKKPVQLPGAYNVNIKLDITSHNEDYTIVEQYERAEGRHSTGGMDELYKARGSAGSATTMVSKGEEDNMAIIKEFMRFKVHMEGSVNGHEFEIEGEGEGRPYEGTQTAKLKVTKGGPLPFAWDILSPQFMYGSKAYVKHPADIPDYLKLSFPEGFKWERVMNFEDGGVVTVTQDSSLQDGEFIYKVKLRGTNFPSDGPVMQKKTMGWEASSERMYPEDGALKGEIKQRLKLKDGGHYDAEVKTTYKAKKPVQLPGAYNVNIKLDITSHNEDYTIVEQYERAEGRHSTGGMDELYKASGSGATNFSLLKQAGDVEENPG**\|<....>\|**PSPAMPEPAKSAPAPKKGSKKAVTKAQKKGGKKRKRSRKESYSIYVYKVLKQVHPDTGISSKAMGIMNSFVNDIFERIAGEASRLAHYNKRSTITSREIQTAVRLLLPGELAKHAVSEGTKAITKYTSAKDPGGGSSRVSKGEELIKENMHMKLYMEGTVDNHHFKCTSEGEGKPYEGTQTMRIKVVEGGPLPFAFDILATSFLYGSKTFINHTQGIPDFFKQSFPEGFTWERVTTYEDGGVLTATQDTSLQDGCLIYNVKIRGVNFTSNGPVMQKKTLGWEAFTETLYPADGGLEGRNDMALKLVGGSHLIANAKTTYRSKKPAKNLKMPGVYYVDYRLERIKEANNETYVEQHEVAVARYCDLPSKLGHKLNGMDELYKGRSPGLNGGSGATNFSLLKQAGDVEENPG | **pC** | **S742** |
| **ePKA-KTR1.2/mCherry.2a.ER/mTagBFP2** | **MEMPEEPANSGHSLPPVYIYSPEYVSICDSLVKVPKRASMVNEDEAPSKRRASLGVSSRLERLTLQSS**GAPVSKGEEDNMAIIKEFMRFKVHMEGSVNGHEFEIEGEGEGRPYEGTQTAKLKVTKGGPLPFAWDILSPQFMYGSKAYVKHPADIPDYLKLSFPEGFKWERVMNFEDGGVVTVTQDSSLQDGEFIYKVKLRGTNFPSDGPVMQKKTMGWEASSERMYPEDGALKGEIKQRLKLKDGGHYDAEVKTTYKAKKPVQLPGAYNVNIKLDITSHNEDYTIVEQYERAEGRHSTGGMDELYKASGSGATNFSLLKQAGDVEENPG**\|<....>\|**PMKLSLVAAMLLLLSAARAVSKGEELIKENMHMKLYMEGTVDNHHFKCTSEGEGKPYEGTQTMRIKVVEGGPLPFAFDILATSFLYGSKTFINHTQGIPDFFKQSFPEGFTWERVTTYEDGGVLTATQDTSLQDGCLIYNVKIRGVNFTSNGPVMQKKTLGWEAFTETLYPADGGLEGRNDMALKLVGGSHLIANAKTTYRSKKPAKNLKMPGVYYVDYRLERIKEANNETYVEQHEVAVARYCDLPSKLGHKLNKDEL | **pLenti** | **YG61** |
| **ePKA-KTR1.4/mCherry.2a.ER-mTagBFP2** | **MEMPEEPANSGHSLPPVYIYSPEYVSICDSLVKVPKRASMVNEEDEAPAKRRASLGVSSRLERLTLQSS**GAPVSKGEEDNMAIIKEFMRFKVHMEGSVNGHEFEIEGEGEGRPYEGTQTAKLKVTKGGPLPFAWDILSPQFMYGSKAYVKHPADIPDYLKLSFPEGFKWERVMNFEDGGVVTVTQDSSLQDGEFIYKVKLRGTNFPSDGPVMQKKTMGWEASSERMYPEDGALKGEIKQRLKLKDGGHYDAEVKTTYKAKKPVQLPGAYNVNIKLDITSHNEDYTIVEQYERAEGRHSTGGMDELYKASGSGATNFSLLKQAGDVEENPG**\|<....>\|**PMKLSLVAAMLLLLSAARAVSKGEELIKENMHMKLYMEGTVDNHHFKCTSEGEGKPYEGTQTMRIKVVEGGPLPFAFDILATSFLYGSKTFINHTQGIPDFFKQSFPEGFTWERVTTYEDGGVLTATQDTSLQDGCLIYNVKIRGVNFTSNGPVMQKKTLGWEAFTETLYPADGGLEGRNDMALKLVGGSHLIANAKTTYRSKKPAKNLKMPGVYYVDYRLERIKEANNETYVEQHEVAVARYCDLPSKLGHKLNKDEL | **pLenti** | **YG65** |
| **ePKA-KTR1.4/tdCherry.2a.ER/mTagBFP2** | **MEMPEEPANSGHSLPPVYIYSPEYVSICDSLVKVPKRASMVNEEDEAPAKRRASLGVSSRLERLTLQSS**GAPVSKGEEDNMAIIKEFMRFKVHMEGSVNGHEFEIEGEGEGRPYEGTQTAKLKVTKGGPLPFAWDILSPQFMYGSKAYVKHPADIPDYLKLSFPEGFKWERVMNFEDGGVVTVTQDSSLQDGEFIYKVKLRGTNFPSDGPVMQKKTMGWEASSERMYPEDGALKGEIKQRLKLKDGGHYDAEVKTTYKAKKPVQLPGAYNVNIKLDITSHNEDYTIVEQYERAEGRHSTGGMDELYKARGSAGSATTMVSKGEEDNMAIIKEFMRFKVHMEGSVNGHEFEIEGEGEGRPYEGTQTAKLKVTKGGPLPFAWDILSPQFMYGSKAYVKHPADIPDYLKLSFPEGFKWERVMNFEDGGVVTVTQDSSLQDGEFIYKVKLRGTNFPSDGPVMQKKTMGWEASSERMYPEDGALKGEIKQRLKLKDGGHYDAEVKTTYKAKKPVQLPGAYNVNIKLDITSHNEDYTIVEQYERAEGRHSTGGMDELYKASGSGATNFSLLKQAGDVEENPG**\|<....>\|**PMKLSLVAAMLLLLSAARAVSKGEELIKENMHMKLYMEGTVDNHHFKCTSEGEGKPYEGTQTMRIKVVEGGPLPFAFDILATSFLYGSKTFINHTQGIPDFFKQSFPEGFTWERVTTYEDGGVLTATQDTSLQDGCLIYNVKIRGVNFTSNGPVMQKKTLGWEAFTETLYPADGGLEGRNDMALKLVGGSHLIANAKTTYRSKKPAKNLKMPGVYYVDYRLERIKEANNETYVEQHEVAVARYCDLPSKLGHKLNKDEL | **pLenti** | **YG67** |
| **ePKA-KTR1.2/mScarlet-I.2a.ER/mTagBFP2** | **MEMPEEPANSGHSLPPVYIYSPEYVSICDSLVKVPKRASMVNEDEAPSKRRASLGVSSRLERLTLQSS**GAPVSKGEAVIKEFMRFKVHMEGSMNGHEFEIEGEGEGRPYEGTQTAKLKVTKGGPLPFSWDILSPQFMYGSRAFIKHPADIPDYYKQSFPEGFKWERVMNFEDGGAVTVTQDTSLEDGTLIYKVKLRGTNFPPDGPVMQKKTMGWEASTERLYPEDGVLKGDIKMALRLKDGGRYLADFKTTYKAKKPVQMPGAYNVDRKLDITSHNEDYTVVEQYERSEGRHSTGGMDELYKASGSGATNFSLLKQAGDVEENPG**\|<....>\|**PMKLSLVAAMLLLLSAARAVSKGEELIKENMHMKLYMEGTVDNHHFKCTSEGEGKPYEGTQTMRIKVVEGGPLPFAFDILATSFLYGSKTFINHTQGIPDFFKQSFPEGFTWERVTTYEDGGVLTATQDTSLQDGCLIYNVKIRGVNFTSNGPVMQKKTLGWEAFTETLYPADGGLEGRNDMALKLVGGSHLIANAKTTYRSKKPAKNLKMPGVYYVDYRLERIKEANNETYVEQHEVAVARYCDLPSKLGHKLNKDEL | **pLenti** | **YG8** |
| **ePKA-KTR1.2/mScarlet-I/BCR.2a.ER/mTagBFP2** | **MEMPEEPANSGHSLPPVYIYSPEYVSICDSLVKVPKRASMVNEDEAPSKRRASLGVSSRLERLTLQSS**GAPVSKGEAVIKEFMRFKVHMEGSMNGHEFEIEGEGEGRPYEGTQTAKLKVTKGGPLPFSWDILSPQFMYGSRAFIKHPADIPDYYKQSFPEGFKWERVMNFEDGGAVTVTQDTSLEDGTLIYKVKLRGTNFPPDGPVMQKKTMGWEASTERLYPEDGVLKGDIKMALRLKDGGRYLADFKTTYKAKKPVQMPGAYNVDRKLDITSHNEDYTVVEQYERSEGRHSTGGMDELYKGSGSAGGSAGGSDIEQELERAKASIRRLEQEVNQERSRMAYLQTLLAKGGSAGGSASGSGATNFSLLKQAGDVEENPG**\|<....>\|**PMKLSLVAAMLLLLSAARAVSKGEELIKENMHMKLYMEGTVDNHHFKCTSEGEGKPYEGTQTMRIKVVEGGPLPFAFDILATSFLYGSKTFINHTQGIPDFFKQSFPEGFTWERVTTYEDGGVLTATQDTSLQDGCLIYNVKIRGVNFTSNGPVMQKKTLGWEAFTETLYPADGGLEGRNDMALKLVGGSHLIANAKTTYRSKKPAKNLKMPGVYYVDYRLERIKEANNETYVEQHEVAVARYCDLPSKLGHKLNKDEL | **pLenti** | **YG18** |
| **ERK-KTR1/tdCherry.2a.H2B/mTagBFP2** | **MKGRKPRDLELPLSPSLLGGQGPERTPGSGTSSGLQAPGPALSPSKRSGLEDPATPSKKPRTPSVSSRLERLTLQSSFQFPS**GAPVSKGEEDNMAIIKEFMRFKVHMEGSVNGHEFEIEGEGEGRPYEGTQTAKLKVTKGGPLPFAWDILSPQFMYGSKAYVKHPADIPDYLKLSFPEGFKWERVMNFEDGGVVTVTQDSSLQDGEFIYKVKLRGTNFPSDGPVMQKKTMGWEASSERMYPEDGALKGEIKQRLKLKDGGHYDAEVKTTYKAKKPVQLPGAYNVNIKLDITSHNEDYTIVEQYERAEGRHSTGGMDELYKARGSAGSATTMVSKGEEDNMAIIKEFMRFKVHMEGSVNGHEFEIEGEGEGRPYEGTQTAKLKVTKGGPLPFAWDILSPQFMYGSKAYVKHPADIPDYLKLSFPEGFKWERVMNFEDGGVVTVTQDSSLQDGEFIYKVKLRGTNFPSDGPVMQKKTMGWEASSERMYPEDGALKGEIKQRLKLKDGGHYDAEVKTTYKAKKPVQLPGAYNVNIKLDITSHNEDYTIVEQYERAEGRHSTGGMDELYKASGSGATNFSLLKQAGDVEENPG**\|<....>\|**PSPAMPEPAKSAPAPKKGSKKAVTKAQKKGGKKRKRSRKESYSIYVYKVLKQVHPDTGISSKAMGIMNSFVNDIFERIAGEASRLAHYNKRSTITSREIQTAVRLLLPGELAKHAVSEGTKAITKYTSAKDPGGGSSRVSKGEELIKENMHMKLYMEGTVDNHHFKCTSEGEGKPYEGTQTMRIKVVEGGPLPFAFDILATSFLYGSKTFINHTQGIPDFFKQSFPEGFTWERVTTYEDGGVLTATQDTSLQDGCLIYNVKIRGVNFTSNGPVMQKKTLGWEAFTETLYPADGGLEGRNDMALKLVGGSHLIANAKTTYRSKKPAKNLKMPGVYYVDYRLERIKEANNETYVEQHEVAVARYCDLPSKLGHKLNGMDELYKGRSPGLNGGSGATNFSLLKQAGDVEENPG | **pC** | **S468** |
| **eERK-KTR1.1/tdCherry.2a.H2B/mTagBFP2** | **MKGRKPRDLELPLSPSLLGGQGPERTPGSGTSSGLQAPGPALSPSKRSGLEDEPATPSKKPRTPSVSSRLERLTLQSSFQFPS**GAPVSKGEEDNMAIIKEFMRFKVHMEGSVNGHEFEIEGEGEGRPYEGTQTAKLKVTKGGPLPFAWDILSPQFMYGSKAYVKHPADIPDYLKLSFPEGFKWERVMNFEDGGVVTVTQDSSLQDGEFIYKVKLRGTNFPSDGPVMQKKTMGWEASSERMYPEDGALKGEIKQRLKLKDGGHYDAEVKTTYKAKKPVQLPGAYNVNIKLDITSHNEDYTIVEQYERAEGRHSTGGMDELYKARGSAGSATTMVSKGEEDNMAIIKEFMRFKVHMEGSVNGHEFEIEGEGEGRPYEGTQTAKLKVTKGGPLPFAWDILSPQFMYGSKAYVKHPADIPDYLKLSFPEGFKWERVMNFEDGGVVTVTQDSSLQDGEFIYKVKLRGTNFPSDGPVMQKKTMGWEASSERMYPEDGALKGEIKQRLKLKDGGHYDAEVKTTYKAKKPVQLPGAYNVNIKLDITSHNEDYTIVEQYERAEGRHSTGGMDELYKASGSGATNFSLLKQAGDVEENPG**\|<....>\|**PSPAMPEPAKSAPAPKKGSKKAVTKAQKKGGKKRKRSRKESYSIYVYKVLKQVHPDTGISSKAMGIMNSFVNDIFERIAGEASRLAHYNKRSTITSREIQTAVRLLLPGELAKHAVSEGTKAITKYTSAKDPGGGSSRVSKGEELIKENMHMKLYMEGTVDNHHFKCTSEGEGKPYEGTQTMRIKVVEGGPLPFAFDILATSFLYGSKTFINHTQGIPDFFKQSFPEGFTWERVTTYEDGGVLTATQDTSLQDGCLIYNVKIRGVNFTSNGPVMQKKTLGWEAFTETLYPADGGLEGRNDMALKLVGGSHLIANAKTTYRSKKPAKNLKMPGVYYVDYRLERIKEANNETYVEQHEVAVARYCDLPSKLGHKLNGMDELYKGRSPGLNGGSGATNFSLLKQAGDVEENPG | **pC** | **S712** |
| **eERK-KTR1.2/tdCherry.2a.H2B/mTagBFP2** | **MKGRKPRDLELPLSPSLLGGQGPERTPGSGTSSGLQAPGPALSPSKRSGLEDDEPATPSKKPRTPSVSSRLERLTLQSSFQFPS**GAPVSKGEEDNMAIIKEFMRFKVHMEGSVNGHEFEIEGEGEGRPYEGTQTAKLKVTKGGPLPFAWDILSPQFMYGSKAYVKHPADIPDYLKLSFPEGFKWERVMNFEDGGVVTVTQDSSLQDGEFIYKVKLRGTNFPSDGPVMQKKTMGWEASSERMYPEDGALKGEIKQRLKLKDGGHYDAEVKTTYKAKKPVQLPGAYNVNIKLDITSHNEDYTIVEQYERAEGRHSTGGMDELYKARGSAGSATTMVSKGEEDNMAIIKEFMRFKVHMEGSVNGHEFEIEGEGEGRPYEGTQTAKLKVTKGGPLPFAWDILSPQFMYGSKAYVKHPADIPDYLKLSFPEGFKWERVMNFEDGGVVTVTQDSSLQDGEFIYKVKLRGTNFPSDGPVMQKKTMGWEASSERMYPEDGALKGEIKQRLKLKDGGHYDAEVKTTYKAKKPVQLPGAYNVNIKLDITSHNEDYTIVEQYERAEGRHSTGGMDELYKASGSGATNFSLLKQAGDVEENPG**\|<....>\|**PSPAMPEPAKSAPAPKKGSKKAVTKAQKKGGKKRKRSRKESYSIYVYKVLKQVHPDTGISSKAMGIMNSFVNDIFERIAGEASRLAHYNKRSTITSREIQTAVRLLLPGELAKHAVSEGTKAITKYTSAKDPGGGSSRVSKGEELIKENMHMKLYMEGTVDNHHFKCTSEGEGKPYEGTQTMRIKVVEGGPLPFAFDILATSFLYGSKTFINHTQGIPDFFKQSFPEGFTWERVTTYEDGGVLTATQDTSLQDGCLIYNVKIRGVNFTSNGPVMQKKTLGWEAFTETLYPADGGLEGRNDMALKLVGGSHLIANAKTTYRSKKPAKNLKMPGVYYVDYRLERIKEANNETYVEQHEVAVARYCDLPSKLGHKLNGMDELYKGRSPGLNGGSGATNFSLLKQAGDVEENPG | **pC** | **S713** |
| **eERK-KTR1.3/tdCherry.2a.H2B/mTagBFP2** | **MKGRKPRDLELPLSPSLLGGQGPERTPGSGTSSGLQAPGPALSPSKRPGLEDDEPATPSKKPRTPSVSSRLERLTLQSSFQFPS**GAPVSKGEEDNMAIIKEFMRFKVHMEGSVNGHEFEIEGEGEGRPYEGTQTAKLKVTKGGPLPFAWDILSPQFMYGSKAYVKHPADIPDYLKLSFPEGFKWERVMNFEDGGVVTVTQDSSLQDGEFIYKVKLRGTNFPSDGPVMQKKTMGWEASSERMYPEDGALKGEIKQRLKLKDGGHYDAEVKTTYKAKKPVQLPGAYNVNIKLDITSHNEDYTIVEQYERAEGRHSTGGMDELYKARGSAGSATTMVSKGEEDNMAIIKEFMRFKVHMEGSVNGHEFEIEGEGEGRPYEGTQTAKLKVTKGGPLPFAWDILSPQFMYGSKAYVKHPADIPDYLKLSFPEGFKWERVMNFEDGGVVTVTQDSSLQDGEFIYKVKLRGTNFPSDGPVMQKKTMGWEASSERMYPEDGALKGEIKQRLKLKDGGHYDAEVKTTYKAKKPVQLPGAYNVNIKLDITSHNEDYTIVEQYERAEGRHSTGGMDELYKASGSGATNFSLLKQAGDVEENPG**\|<....>\|**PSPAMPEPAKSAPAPKKGSKKAVTKAQKKGGKKRKRSRKESYSIYVYKVLKQVHPDTGISSKAMGIMNSFVNDIFERIAGEASRLAHYNKRSTITSREIQTAVRLLLPGELAKHAVSEGTKAITKYTSAKDPGGGSSRVSKGEELIKENMHMKLYMEGTVDNHHFKCTSEGEGKPYEGTQTMRIKVVEGGPLPFAFDILATSFLYGSKTFINHTQGIPDFFKQSFPEGFTWERVTTYEDGGVLTATQDTSLQDGCLIYNVKIRGVNFTSNGPVMQKKTLGWEAFTETLYPADGGLEGRNDMALKLVGGSHLIANAKTTYRSKKPAKNLKMPGVYYVDYRLERIKEANNETYVEQHEVAVARYCDLPSKLGHKLNGMDELYKGRSPGLNGGSGATNFSLLKQAGDVEENPG | **pC** | **S765** |
| **eERK-KTR1.2/mScarlet-I.2a.ER/mTagBFP2** | **MKGRKPRDLELPLSPSLLGGQGPERTPGSGTSSGLQAPGPALSPSKRSGLEDDEPATPSKKPRTPSVSSRLERLTLQSSFQFPS**GAPVSKGEAVIKEFMRFKVHMEGSMNGHEFEIEGEGEGRPYEGTQTAKLKVTKGGPLPFSWDILSPQFMYGSRAFIKHPADIPDYYKQSFPEGFKWERVMNFEDGGAVTVTQDTSLEDGTLIYKVKLRGTNFPPDGPVMQKKTMGWEASTERLYPEDGVLKGDIKMALRLKDGGRYLADFKTTYKAKKPVQMPGAYNVDRKLDITSHNEDYTVVEQYERSEGRHSTGGMDELYKASGSGATNFSLLKQAGDVEENPG**\|<....>\|**PMKLSLVAAMLLLLSAARAVSKGEELIKENMHMKLYMEGTVDNHHFKCTSEGEGKPYEGTQTMRIKVVEGGPLPFAFDILATSFLYGSKTFINHTQGIPDFFKQSFPEGFTWERVTTYEDGGVLTATQDTSLQDGCLIYNVKIRGVNFTSNGPVMQKKTLGWEAFTETLYPADGGLEGRNDMALKLVGGSHLIANAKTTYRSKKPAKNLKMPGVYYVDYRLERIKEANNETYVEQHEVAVARYCDLPSKLGHKLNKDEL | **pLenti** | **S940** |
| **eERK-KTR1.2/mScarlet-I/BCR.2a.ER/mTagBFP2** | **MKGRKPRDLELPLSPSLLGGQGPERTPGSGTSSGLQAPGPALSPSKRSGLEDDEPATPSKKPRTPSVSSRLERLTLQSSFQFPS**GAPVSKGEAVIKEFMRFKVHMEGSMNGHEFEIEGEGEGRPYEGTQTAKLKVTKGGPLPFSWDILSPQFMYGSRAFIKHPADIPDYYKQSFPEGFKWERVMNFEDGGAVTVTQDTSLEDGTLIYKVKLRGTNFPPDGPVMQKKTMGWEASTERLYPEDGVLKGDIKMALRLKDGGRYLADFKTTYKAKKPVQMPGAYNVDRKLDITSHNEDYTVVEQYERSEGRHSTGGMDELYKGSGSAGGSAGGSDIEQELERAKASIRRLEQEVNQERSRMAYLQTLLAKGGSAGGSASGSGATNFSLLKQAGDVEENPG**\|<....>\|**PMKLSLVAAMLLLLSAARAVSKGEELIKENMHMKLYMEGTVDNHHFKCTSEGEGKPYEGTQTMRIKVVEGGPLPFAFDILATSFLYGSKTFINHTQGIPDFFKQSFPEGFTWERVTTYEDGGVLTATQDTSLQDGCLIYNVKIRGVNFTSNGPVMQKKTLGWEAFTETLYPADGGLEGRNDMALKLVGGSHLIANAKTTYRSKKPAKNLKMPGVYYVDYRLERIKEANNETYVEQHEVAVARYCDLPSKLGHKLNKDEL | **pLenti** | **YG21** |
| **ePKA-KTR1.2/tdCherry.2a.3xNLS/mTagBFP2** | **MEMPEEPANSGHSLPPVYIYSPEYVSICDSLVKVPKRASMVNEDEAPSKRRASLGVSSRLERLTLQSS**GAPVSKGEEDNMAIIKEFMRFKVHMEGSVNGHEFEIEGEGEGRPYEGTQTAKLKVTKGGPLPFAWDILSPQFMYGSKAYVKHPADIPDYLKLSFPEGFKWERVMNFEDGGVVTVTQDSSLQDGEFIYKVKLRGTNFPSDGPVMQKKTMGWEASSERMYPEDGALKGEIKQRLKLKDGGHYDAEVKTTYKAKKPVQLPGAYNVNIKLDITSHNEDYTIVEQYERAEGRHSTGGMDELYKARGSAGSATTMVSKGEEDNMAIIKEFMRFKVHMEGSVNGHEFEIEGEGEGRPYEGTQTAKLKVTKGGPLPFAWDILSPQFMYGSKAYVKHPADIPDYLKLSFPEGFKWERVMNFEDGGVVTVTQDSSLQDGEFIYKVKLRGTNFPSDGPVMQKKTMGWEASSERMYPEDGALKGEIKQRLKLKDGGHYDAEVKTTYKAKKPVQLPGAYNVNIKLDITSHNEDYTIVEQYERAEGRHSTGGMDELYKASGSGATNFSLLKQAGDVEENPG**\|<....>\|**PSPAPKKKRKVGDGGCPKKKRKVGDGGCPKKKRKVGDSSRVSKGEELIKENMHMKLYMEGTVDNHHFKCTSEGEGKPYEGTQTMRIKVVEGGPLPFAFDILATSFLYGSKTFINHTQGIPDFFKQSFPEGFTWERVTTYEDGGVLTATQDTSLQDGCLIYNVKIRGVNFTSNGPVMQKKTLGWEAFTETLYPADGGLEGRNDMALKLVGGSHLIANAKTTYRSKKPAKNLKMPGVYYVDYRLERIKEANNETYVEQHEVAVARYCDLPSKLGHKLNGMDELYKGRSPGLNGGSGATNFSLLKQAGDVEENPG | **pC** | **S381** |
| **ePKA-KTR1.2/tdCherry.2a.ER/mTagBFP2** | **MEMPEEPANSGHSLPPVYIYSPEYVSICDSLVKVPKRASMVNEDEAPSKRRASLGVSSRLERLTLQSS**GAPVSKGEEDNMAIIKEFMRFKVHMEGSVNGHEFEIEGEGEGRPYEGTQTAKLKVTKGGPLPFAWDILSPQFMYGSKAYVKHPADIPDYLKLSFPEGFKWERVMNFEDGGVVTVTQDSSLQDGEFIYKVKLRGTNFPSDGPVMQKKTMGWEASSERMYPEDGALKGEIKQRLKLKDGGHYDAEVKTTYKAKKPVQLPGAYNVNIKLDITSHNEDYTIVEQYERAEGRHSTGGMDELYKARGSAGSATTMVSKGEEDNMAIIKEFMRFKVHMEGSVNGHEFEIEGEGEGRPYEGTQTAKLKVTKGGPLPFAWDILSPQFMYGSKAYVKHPADIPDYLKLSFPEGFKWERVMNFEDGGVVTVTQDSSLQDGEFIYKVKLRGTNFPSDGPVMQKKTMGWEASSERMYPEDGALKGEIKQRLKLKDGGHYDAEVKTTYKAKKPVQLPGAYNVNIKLDITSHNEDYTIVEQYERAEGRHSTGGMDELYKASGSGATNFSLLKQAGDVEENPG**\|<....>\|**PMKLSLVAAMLLLLSAARAVSKGEELIKENMHMKLYMEGTVDNHHFKCTSEGEGKPYEGTQTMRIKVVEGGPLPFAFDILATSFLYGSKTFINHTQGIPDFFKQSFPEGFTWERVTTYEDGGVLTATQDTSLQDGCLIYNVKIRGVNFTSNGPVMQKKTLGWEAFTETLYPADGGLEGRNDMALKLVGGSHLIANAKTTYRSKKPAKNLKMPGVYYVDYRLERIKEANNETYVEQHEVAVARYCDLPSKLGHKLNKDEL | **pLenti** | **YG63** |
| **ePKA-KTR1.2/tdTomato.2a.ER/mTagBFP2** | **MEMPEEPANSGHSLPPVYIYSPEYVSICDSLVKVPKRASMVNEDEAPSKRRASLGVSSRLERLTLQSS**GAPVSKGEEVIKEFMRFKVRMEGSMNGHEFEIEGEGEGRPYEGTQTAKLKVTKGGPLPFAWDILSPQFMYGSKAYVKHPADIPDYKKLSFPEGFKWERVMNFEDGGLVTVTQDSSLQDGTLIYKVKMRGTNFPPDGPVMQKKTMGWEASTERLYPRDGVLKGEIHQALKLKDGGHYLVEFKTIYMAKKPVQLPGYYYVDTKLDITSHNEDYTIVEQYERSEGRHHLFLGHGTGSTGSGSSGTASSEDNNMAVIKEFMRFKVRMEGSMNGHEFEIEGEGEGRPYEGTQTAKLKVTKGGPLPFAWDILSPQFMYGSKAYVKHPADIPDYKKLSFPEGFKWERVMNFEDGGLVTVTQDSSLHDGTLIYKVKMRGTNFPPDGPVMQKKTMGWEASTERLYPRDGVLKGDIHQALKLKDGGHYLVEFKTIYMAKKPVQLPGYYYVDTKLDITSHNEDYTIVEQYERSEGRHHLFLYGMDELYKASGSGATNFSLLKQAGDVEENPG**\|<....>\|**PMKLSLVAAMLLLLSAARAVSKGEELIKENMHMKLYMEGTVDNHHFKCTSEGEGKPYEGTQTMRIKVVEGGPLPFAFDILATSFLYGSKTFINHTQGIPDFFKQSFPEGFTWERVTTYEDGGVLTATQDTSLQDGCLIYNVKIRGVNFTSNGPVMQKKTLGWEAFTETLYPADGGLEGRNDMALKLVGGSHLIANAKTTYRSKKPAKNLKMPGVYYVDYRLERIKEANNETYVEQHEVAVARYCDLPSKLGHKLNKDEL | **pLenti** | **YG50** |
| **eERK-KTR1.2/emiRFP670.2a.ER/mTagBFP2** | **MKGRKPRDLELPLSPSLLGGQGPERTPGSGTSSGLQAPGPALSPSKRSGLEDDEPATPSKKPRTPSVSSRLERLTLQSSFQFPS**GAPMAEGSVARQPDLLTCEHEEIHLAGSIQPHGALLVVSEHDHRVIQASANAAEFLNLGSVLGVPLAEIDGDLLIKILPHLDPTAEGMPVAVRCRIGNPSTEYCGLMHRPPEGGLIIELERAGPSIDLSGTLAPALERIRTAGSLRALCDDTVLLFQQCTGYDRVMVYRFDEQGHGLVFSECHVPGLESYFGNRYPSSTVPQMARQLYVRQRVRVLVDVTYQPVPLEPRLSPLTGRDLDMSGCFLRSMSPCHLQFLKDMGVRATLAVSLVVGGKLWGLVVCHHYLPRFIRFELRAICKRLAERIATRITALESLYKASGSGATNFSLLKQAGDVEENPG**\|<....>\|**PMKLSLVAAMLLLLSAARAVSKGEELIKENMHMKLYMEGTVDNHHFKCTSEGEGKPYEGTQTMRIKVVEGGPLPFAFDILATSFLYGSKTFINHTQGIPDFFKQSFPEGFTWERVTTYEDGGVLTATQDTSLQDGCLIYNVKIRGVNFTSNGPVMQKKTLGWEAFTETLYPADGGLEGRNDMALKLVGGSHLIANAKTTYRSKKPAKNLKMPGVYYVDYRLERIKEANNETYVEQHEVAVARYCDLPSKLGHKLNKDEL | **pLenti** | **YG6** |
| **ePKA-KTR1.4/tdTomato.2a.ER/mTagBFP2** | **MEMPEEPANSGHSLPPVYIYSPEYVSICDSLVKVPKRASMVNEEDEAPAKRRASLGVSSRLERLTLQSS**GAPVSKGEEVIKEFMRFKVRMEGSMNGHEFEIEGEGEGRPYEGTQTAKLKVTKGGPLPFAWDILSPQFMYGSKAYVKHPADIPDYKKLSFPEGFKWERVMNFEDGGLVTVTQDSSLQDGTLIYKVKMRGTNFPPDGPVMQKKTMGWEASTERLYPRDGVLKGEIHQALKLKDGGHYLVEFKTIYMAKKPVQLPGYYYVDTKLDITSHNEDYTIVEQYERSEGRHHLFLGHGTGSTGSGSSGTASSEDNNMAVIKEFMRFKVRMEGSMNGHEFEIEGEGEGRPYEGTQTAKLKVTKGGPLPFAWDILSPQFMYGSKAYVKHPADIPDYKKLSFPEGFKWERVMNFEDGGLVTVTQDSSLHDGTLIYKVKMRGTNFPPDGPVMQKKTMGWEASTERLYPRDGVLKGDIHQALKLKDGGHYLVEFKTIYMAKKPVQLPGYYYVDTKLDITSHNEDYTIVEQYERSEGRHHLFLYGMDELYKASGSGATNFSLLKQAGDVEENPG**\|<....>\|**PMKLSLVAAMLLLLSAARAVSKGEELIKENMHMKLYMEGTVDNHHFKCTSEGEGKPYEGTQTMRIKVVEGGPLPFAFDILATSFLYGSKTFINHTQGIPDFFKQSFPEGFTWERVTTYEDGGVLTATQDTSLQDGCLIYNVKIRGVNFTSNGPVMQKKTLGWEAFTETLYPADGGLEGRNDMALKLVGGSHLIANAKTTYRSKKPAKNLKMPGVYYVDYRLERIKEANNETYVEQHEVAVARYCDLPSKLGHKLNKDEL | **pLenti** | **YG115** |
| **PKA-KTR2/mCherry.2a.H2B/mTagBFP2** | **MVLRRASLGKRRASLGKRRASLGVDQLRLERLQIDE**GAPVSKGEEDNMAIIKEFMRFKVHMEGSVNGHEFEIEGEGEGRPYEGTQTAKLKVTKGGPLPFAWDILSPQFMYGSKAYVKHPADIPDYLKLSFPEGFKWERVMNFEDGGVVTVTQDSSLQDGEFIYKVKLRGTNFPSDGPVMQKKTMGWEASSERMYPEDGALKGEIKQRLKLKDGGHYDAEVKTTYKAKKPVQLPGAYNVNIKLDITSHNEDYTIVEQYERAEGRHSTGGMDELYKASGSGATNFSLLKQAGDVEENPG**\|<....>\|**PSPAMPEPAKSAPAPKKGSKKAVTKAQKKGGKKRKRSRKESYSIYVYKVLKQVHPDTGISSKAMGIMNSFVNDIFERIAGEASRLAHYNKRSTITSREIQTAVRLLLPGELAKHAVSEGTKAITKYTSAKDPGGGSSRVSKGEELIKENMHMKLYMEGTVDNHHFKCTSEGEGKPYEGTQTMRIKVVEGGPLPFAFDILATSFLYGSKTFINHTQGIPDFFKQSFPEGFTWERVTTYEDGGVLTATQDTSLQDGCLIYNVKIRGVNFTSNGPVMQKKTLGWEAFTETLYPADGGLEGRNDMALKLVGGSHLIANAKTTYRSKKPAKNLKMPGVYYVDYRLERIKEANNETYVEQHEVAVARYCDLPSKLGHKLNGMDELYKGRSPGLNGGSGATNFSLLKQAGDVEENPG | **pC** | **S737** |
| **PKA-KTR2/tdCherry.2a.H2B/mTagBFP2** | **MVLRRASLGKRRASLGKRRASLGVDQLRLERLQIDE**GAPVSKGEEDNMAIIKEFMRFKVHMEGSVNGHEFEIEGEGEGRPYEGTQTAKLKVTKGGPLPFAWDILSPQFMYGSKAYVKHPADIPDYLKLSFPEGFKWERVMNFEDGGVVTVTQDSSLQDGEFIYKVKLRGTNFPSDGPVMQKKTMGWEASSERMYPEDGALKGEIKQRLKLKDGGHYDAEVKTTYKAKKPVQLPGAYNVNIKLDITSHNEDYTIVEQYERAEGRHSTGGMDELYKARGSAGSATTMVSKGEEDNMAIIKEFMRFKVHMEGSVNGHEFEIEGEGEGRPYEGTQTAKLKVTKGGPLPFAWDILSPQFMYGSKAYVKHPADIPDYLKLSFPEGFKWERVMNFEDGGVVTVTQDSSLQDGEFIYKVKLRGTNFPSDGPVMQKKTMGWEASSERMYPEDGALKGEIKQRLKLKDGGHYDAEVKTTYKAKKPVQLPGAYNVNIKLDITSHNEDYTIVEQYERAEGRHSTGGMDELYKASGSGATNFSLLKQAGDVEENPG**\|<....>\|**PSPAMPEPAKSAPAPKKGSKKAVTKAQKKGGKKRKRSRKESYSIYVYKVLKQVHPDTGISSKAMGIMNSFVNDIFERIAGEASRLAHYNKRSTITSREIQTAVRLLLPGELAKHAVSEGTKAITKYTSAKDPGGGSSRVSKGEELIKENMHMKLYMEGTVDNHHFKCTSEGEGKPYEGTQTMRIKVVEGGPLPFAFDILATSFLYGSKTFINHTQGIPDFFKQSFPEGFTWERVTTYEDGGVLTATQDTSLQDGCLIYNVKIRGVNFTSNGPVMQKKTLGWEAFTETLYPADGGLEGRNDMALKLVGGSHLIANAKTTYRSKKPAKNLKMPGVYYVDYRLERIKEANNETYVEQHEVAVARYCDLPSKLGHKLNGMDELYKGRSPGLNGGSGATNFSLLKQAGDVEENPG | **pC** | **S738** |
| **PKA-KTR3/mCherry.2a.ER/mTagBFP2** | **MEMPEEPANSGHSLPPVYIYSPEYSIFDSLVKVKRNPTVVNEDEAPSKRKRSLPISSRLERLTLQSS**GAPVSKGEEDNMAIIKEFMRFKVHMEGSVNGHEFEIEGEGEGRPYEGTQTAKLKVTKGGPLPFAWDILSPQFMYGSKAYVKHPADIPDYLKLSFPEGFKWERVMNFEDGGVVTVTQDSSLQDGEFIYKVKLRGTNFPSDGPVMQKKTMGWEASSERMYPEDGALKGEIKQRLKLKDGGHYDAEVKTTYKAKKPVQLPGAYNVNIKLDITSHNEDYTIVEQYERAEGRHSTGGMDELYKASGSGATNFSLLKQAGDVEENPG**\|<....>\|**PMKLSLVAAMLLLLSAARAVSKGEELIKENMHMKLYMEGTVDNHHFKCTSEGEGKPYEGTQTMRIKVVEGGPLPFAFDILATSFLYGSKTFINHTQGIPDFFKQSFPEGFTWERVTTYEDGGVLTATQDTSLQDGCLIYNVKIRGVNFTSNGPVMQKKTLGWEAFTETLYPADGGLEGRNDMALKLVGGSHLIANAKTTYRSKKPAKNLKMPGVYYVDYRLERIKEANNETYVEQHEVAVARYCDLPSKLGHKLNKDEL | **pLenti** | **YG69** |
| **PKA-KTR3/tdCherry.2a.ER/mTagBFP2** | **MEMPEEPANSGHSLPPVYIYSPEYSIFDSLVKVKRNPTVVNEDEAPSKRKRSLPISSRLERLTLQSS**GAPVSKGEEDNMAIIKEFMRFKVHMEGSVNGHEFEIEGEGEGRPYEGTQTAKLKVTKGGPLPFAWDILSPQFMYGSKAYVKHPADIPDYLKLSFPEGFKWERVMNFEDGGVVTVTQDSSLQDGEFIYKVKLRGTNFPSDGPVMQKKTMGWEASSERMYPEDGALKGEIKQRLKLKDGGHYDAEVKTTYKAKKPVQLPGAYNVNIKLDITSHNEDYTIVEQYERAEGRHSTGGMDELYKARGSAGSATTMVSKGEEDNMAIIKEFMRFKVHMEGSVNGHEFEIEGEGEGRPYEGTQTAKLKVTKGGPLPFAWDILSPQFMYGSKAYVKHPADIPDYLKLSFPEGFKWERVMNFEDGGVVTVTQDSSLQDGEFIYKVKLRGTNFPSDGPVMQKKTMGWEASSERMYPEDGALKGEIKQRLKLKDGGHYDAEVKTTYKAKKPVQLPGAYNVNIKLDITSHNEDYTIVEQYERAEGRHSTGGMDELYKASGSGATNFSLLKQAGDVEENPG**\|<....>\|**PMKLSLVAAMLLLLSAARAVSKGEELIKENMHMKLYMEGTVDNHHFKCTSEGEGKPYEGTQTMRIKVVEGGPLPFAFDILATSFLYGSKTFINHTQGIPDFFKQSFPEGFTWERVTTYEDGGVLTATQDTSLQDGCLIYNVKIRGVNFTSNGPVMQKKTLGWEAFTETLYPADGGLEGRNDMALKLVGGSHLIANAKTTYRSKKPAKNLKMPGVYYVDYRLERIKEANNETYVEQHEVAVARYCDLPSKLGHKLNKDEL | **pLenti** | **YG71** |
| **ERK-KTR1/emiRFP670.2a.ER/mTagBFP2** | **MKGRKPRDLELPLSPSLLGGQGPERTPGSGTSSGLQAPGPALSPSKRSGLEDPATPSKKPRTPSVSSRLERLTLQSSFQFPS**GAPMAEGSVARQPDLLTCEHEEIHLAGSIQPHGALLVVSEHDHRVIQASANAAEFLNLGSVLGVPLAEIDGDLLIKILPHLDPTAEGMPVAVRCRIGNPSTEYCGLMHRPPEGGLIIELERAGPSIDLSGTLAPALERIRTAGSLRALCDDTVLLFQQCTGYDRVMVYRFDEQGHGLVFSECHVPGLESYFGNRYPSSTVPQMARQLYVRQRVRVLVDVTYQPVPLEPRLSPLTGRDLDMSGCFLRSMSPCHLQFLKDMGVRATLAVSLVVGGKLWGLVVCHHYLPRFIRFELRAICKRLAERIATRITALESLYKASGSGATNFSLLKQAGDVEENPG**\|<....>\|**PMKLSLVAAMLLLLSAARAVSKGEELIKENMHMKLYMEGTVDNHHFKCTSEGEGKPYEGTQTMRIKVVEGGPLPFAFDILATSFLYGSKTFINHTQGIPDFFKQSFPEGFTWERVTTYEDGGVLTATQDTSLQDGCLIYNVKIRGVNFTSNGPVMQKKTLGWEAFTETLYPADGGLEGRNDMALKLVGGSHLIANAKTTYRSKKPAKNLKMPGVYYVDYRLERIKEANNETYVEQHEVAVARYCDLPSKLGHKLNKDEL | **pLenti** | **YG45** |

*Table S2. List of plasmid numbers and the ORF they express.*

| **Name** | **Description (ORF expressed)** | **vector backbone** | **promoter** |
| --- | --- | --- | --- |
| S739 | PKA-KTR1/mCherry-2a-H2B/mTagBFP2 | pC (plasmid) | CMV |
| S777 | mCherry/PKA-KTR2-2a-H2B/mTagBFP2 | pC (plasmid) | CMV |
| S740 | PKA-KTR1/tdCherry-2a-H2B/mTagBFP2 | pC (plasmid) | CMV |
| S738 | PKA-KTR2/tdCherry-2a-H2B-mTagBFP2 | pC (plasmid) | CMV |
| S741 | ePKA-KTR1.1/tdCherry-2a-H2B/mTagBFP2 | pC (plasmid) | CMV |
| S467 | ePKA-KTR1.2/tdCherry-2a-H2B/mTagBFP2 | pC (plasmid) | CMV |
| S514 | ePKA-KTR1.3/tdCherry-2a-H2B/mTagBFP2 | pC (plasmid) | CMV |
| S515 | ePKA-KTR1.4/tdCherry-2a-H2B/mTagBFP2 | pC (plasmid) | CMV |
| S742 | ePKA-KTR1.5/tdCherry-2a-H2B/mTagBFP2 | pC (plasmid) | CMV |
| YG63 | ePKA-KTR1.2/tdCherry-2a-ER/mTagBFP2 | pLenti (lentiviral vector) | CMV |
| S381 | ePKA-KTR1.2/tdCherry-2a-3xNLS/mTagBFP2 | pC (plasmid) | CMV |
| YG61 | ePKA-KTR1.2/mCherry-2a-ER/mTagBFP2 | pLenti (lentiviral vector) | CMV |
| YG65 | ePKA-KTR1.4/mCherry-2a-ER/mTagBFP2 | pLenti (lentiviral vector) | CMV |
| YG67 | ePKA-KTR1.4/tdCherry-2a-ER/mTagBFP2 | pLenti (lentiviral vector) | CMV |
| YG8 | ePKA-KTR1.2/mScarlet-2a-ER/mTagBFP2 | pLenti (lentiviral vector) | CMV |
| YG18 | ePKA-KTR1.2/mScarlet/BCR-2a-ER/mTagBFP2 | pLenti (lentiviral vector) | CMV |
| S468 | ERK-KTR1/tdCherry-2a-H2B/mTagBFP2 | pC (plasmid) | CMV |
| S712 | eERK-KTR1.1/tdCherry-2a-H2B/mTagBFP2 | pC (plasmid) | CMV |
| S713 | eERK-KTR1.2/tdCherry-2a-H2B/mTagBFP2 | pC (plasmid) | CMV |
| S765 | eERK-KTR1.3/tdCherry-2a-H2B/mTagBFP2 | pC (plasmid) | CMV |
| S940 | eERK-KTR1.2/mScarlet-2a-ER/mTagBFP2 | pLenti (lentiviral vector) | CMV |
| YG21 | eERK-KTR1.2/mScarlet/BCR-2a-ER/mTagBFP2 | pLenti (lentiviral vector) | CMV |
| YG6 | eERK-KTR1.2/emiRFP670-2a-ER/mTagBFP2 | pLenti (lentiviral vector) | CMV |
| YG45 | ERK-KTR1/emiRFP670-2a-ER/mTagBFP2 | pLenti (lentiviral vector) | CMV |
| YG50 | ePKA-KTR1.2/tdTomato-2a-ER/mTagBFP2 | pLenti (lentiviral vector) | CMV |
| YG114 | PKA-KTR3/tdTomato-2a-ER/mTagBFP2 | pLenti (lentiviral vector) | CMV |
| YG115 | ePKA-KTR1.4/tdTomato-2a-ER/mTagBFP2 | pLenti (lentiviral vector) | CMV |
| S1137 | jGCaMP8s | Lentiviral | CMV |

**Reagents and Tools Table**

*Instructions: Please complete the relevant fields below, adding rows as needed. The following page provides an example of a completed table and additional instruction for entering your data in the table.*

| **Reagent/Resource** | **Reference or Source** | **Identifier or Catalog Number** |
| --- | --- | --- |
|  |  |  |
| **Experimental Models** |  |  |
| HEK-293 cells (H. sapiens) | ATCC | CRL-1573 |
| Dorsal root ganglion neurons | C57BL/6 (Jackson Lab) | 000664 |
| **Recombinant DNA** |  |  |
| psPAX2 | Addgene | 12260 |
| VSV.G | Addgene | 14888 |
| PKA-KTR1/tdCherry.2a.H2B/mTagBFP2 | This study | N/A |
| PKA-KTR1/mCherry.2a.H2B/mTagBFP2 | This study | N/A |
| ePKA-KTR1.1/tdCherry.2a.H2B/mTagBFP2 | This study | N/A |
| ePKA-KTR1.2/tdCherry.2a.H2B/mTagBFP2 | This study | N/A |
| ePKA-KTR1.3/tdCherry.2a.H2B/mTagBFP2 | This study | N/A |
| ePKA-KTR1.4/tdCherry.2a.H2B/mTagBFP2 | This study | N/A |
| ePKA-KTR1.5/tdCherry.2a.H2B/mTagBFP2 | This study | N/A |
| ePKA-KTR1.2/mCherry.2a.ER/mTagBFP2 | This study | N/A |
| ePKA-KTR1.4/mCherry.2a.ER/mTagBFP2 | This study | N/A |
| ePKA-KTR1.4/tdCherry.2a.ER/mTagBFP2 | This study | N/A |
| ePKA-KTR1.2/mScarlet-I.2a.ER/mTagBFP2 | This study | N/A |
| ePKA-KTR1.2/mScarlet-I/BCR.2a.ER/mTagBFP2 | This study | N/A |
| ERK-KTR1/tdCherry.2a.H2B/mTagBFP2 | This study | N/A |
| eERK-KTR1.1/tdCherry.2a.H2B/mTagBFP2 | This study | N/A |
| eERK-KTR1.2/tdCherry.2a.H2B/mTagBFP2 | This study | N/A |
| eERK-KTR1.3/tdCherry.2a.H2B/mTagBFP2 | This study | N/A |
| eERK-KTR1.2/mScarlet-I.2a.ER/mTagBFP2 | This study | N/A |
| eERK-KTR1.2/mScarlet-I/BCR.2a.ER/mTagBFP2 | This study | N/A |
| ePKA-KTR1.2/tdCherry.2a.3xNLS/mTagBFP2 | This study | N/A |
| ePKA-KTR1.2/tdCherry.2a.ER/mTagBFP2 | This study | N/A |
| ePKA-KTR1.2/tdTomato.2a.ER/mTagBFP2 | This study | N/A |
| eERK-KTR1.2/emiRFP670.2a.ER/mTagBFP2 | This study | N/A |
| ePKA-KTR1.4/tdTomato.2a.ER/mTagBFP2 | This study | N/A |
| PKA-KTR2/mCherry.2a.H2B/mTagBFP2 | This study | N/A |
| PKA-KTR2/tdCherry.2a.H2B/mTagBFP2 | This study | N/A |
| PKA-KTR3/mCherry.2a.ER/mTagBFP2 | This study | N/A |
| PKA-KTR3/tdCherry.2a.ER/mTagBFP2 | This study | N/A |
| ERK-KTR1/emiRFP670.2a.ER/mTagBFP2 | This study | N/A |
| Additional plasmids and more information | This study | Table S1 |
| **Antibodies** |  |  |
| Rabbit anti-phospho-ERK1/2 | Cell Signaling Technology | 9101S |
| Rabbit anti-phospho-PKA R2 (Ser99) | Abcam | ab32390 |
| Goat anti-rabbit Alexa488 | Jackson ImmunoResearch | AB_2338046 |
| **Oligonucleotides and other sequence-based reagents** |  |  |
| Geneblocks and primers | Integrated DNA Technologies | Table S1 |
| **Chemicals, Enzymes and other reagents** |  |  |
| Phusion High-Fidelity DNA Polymerase | ThermoFisher | F530L |
| T4 DNA Ligase (5 U/μL) | ThermoFisher | EL0011 |
| Petri Dishes | Falcon | 351029 |
| DMEM, high glucose | ThermoFisher | 11965118 |
| Trypsin-EDTA (0.25%), phenol red | ThermoFisher | 25200114 |
| Fetal Bovine Serum | ThermoFisher | 16000044 |
| 24-well cell culture plates | Denville | 1156F00 |
| TC-treated Culture Dish (100 mm) | Corning | 430167 |
| Hanks' Balanced Salt Solution (HBSS) | ThermoFisher | 14175103 |
| 96 Well Black Plate, Coverglass Bottom, CC2 Surface | ThermoFisher | 160376 |
| µ-Slide 8 Well high Glass Bottom | Ibidi | 80807 |
| µ-Slide 18 Well Glass Bottom | Ibidi | 81817 |
| A23187 | Sigma-Aldrich | C7522 |
| ATP | Sigma-Aldrich | A26209 |
| Forskolin | Cayman Chemical | 11018 |
| H89 | MedChemExpress | HY-15979A |
| EGF | Gibco | PHG0311L |
| VX-11e | SelleckChem | S7709 |
| leptomycin B | Sigma-Aldrich | L2913 |
| Phorbol 12-myristate 13-acetate | Sigma-Aldrich | P1585 |
| Neurobasal™ Plus Medium | Gibco | A3582901 |
| B-27™ Plus Supplement | Gibco | A3582801 |
| Poly-L-lysine solution | Sigma-Aldrich | P8920-100ML |
| Laminin | Sigma-Aldrich | L2020 |
| Papain, suspension | Worthington | LS003126 |
| Bovine Serum Albumin | Sigma-Aldrich | A-9418 |
| TM Liberase | Roche | 5401119001 |
| TL Liberase | Roche | 5401020001 |
| Pen/Strep (100X) | ThermoFisher | 15140122 |
| Lipofectamine™ 3000 | ThermoFisher | L3000015 |
| Open-Top Thinwall Ultra-Clear Tube | Beckman-Coulter | C14292 |
| 50 mL Luer-Lok™ Syringe | BD | 309653 |
| Syringe Filter (0.45 μm) | Millipore | SLHVR33RS |
| 8-mm cover glasses | Electron Microscopy Sciences | 7229608 |
| QIAprep Spin Miniprep Kit | Qiagen | 27106 |
| Wizard® Plus Midipreps DNA Purification System | Promega | A7640 |
| Ampicillin sodium salt | Sigma-Aldrich | A8351 |
| **Software** |  |  |
| GraphPad Prism 10 | https://www.graphpad.com/ |  |
| SnapGene 7 | https://www.snapgene.com/ |  |
| ImageJ (Fiji) | https://fiji.sc/ |  |
| NIS Elements Advanced Research v4.60 | https://www.microscope.healthcare.nikon.com/products/software/nis-elements |  |
| EVOS M7000 Cell Imaging System Software | https://www.thermofisher.com/us/en/home/technical-resources/software-downloads/evos-m7000-imaging-system-software-download.html |  |
| **Other** |  |  |
| EVOS M7000 Imaging System | ThermoFisher | AMF7000 |
| EVOS Onstage Incubator | ThermoFisher | AMC2000 |
| Optima L-90K ultracentrifuge | Beckman-Coulter |  |
| SW-32 Ti rotor | Beckman-Coulter |  |
| Sony MA900 Cell Sorter | Sony |  |

**REFERENCES**

1. Kim, A. K., Wu, H. D., andInoue, T. (2020) Rational Design of a Protein Kinase A Nuclear-cytosol Translocation Reporter Sci Rep **10**, 9365 10.1038/s41598-020-66349-3

2. Kudo, T., Lane, K., and Covert, M. W. (2022) A multiplexed epitope barcoding strategy that enables dynamic cellular phenotypic screens Cell Syst **13**, 376-387 e378 10.1016/j.cels.2022.02.006

3. Guo, C., Fordjour, F. K., Tsai, S. J., Morrell, J. C., andGould, S. J. (2021) Choice of selectable marker affects recombinant protein expression in cells and exosomes J Biol Chem 100838 10.1016/j.jbc.2021.100838
